# Supplementary material for: Genome-wide identification, characterization and gene expression of BES1 transcription factor family in grapevine (Vitis vinifera L.)
Source: Sci Rep. 2023 Jan 5;13:240. doi: 10.1038/s41598-022-24407-y (PMC9816167; doi:10.1038/s41598-022-24407-y)
Supplement: Supplementary file 3 — Supplementary Information. [file 41598_2022_24407_MOESM3_ESM.zip › Vvi_Vvi/Vitis_vinifera.PN40024.v4.dna_sm.toplevel.fa.vs.Vitis_vinifera.PN40024.v4.dna_sm.toplevel.fa.html/11.html]

|  |  |  |  |  |  |  |  |  |  |  |  |  |  |
| --- | --- | --- | --- | --- | --- | --- | --- | --- | --- | --- | --- | --- | --- |
| Duplication depth | Reference chromosome | Collinear blocks | | | | | | | | | | | |
| 0 | Vitvi11g04000\_t001 |  |  |  |  |  |  |  |  |  |  |  |  |
| 0 | Vitvi11g04001\_t001 |  |  |  |  |  |  |  |  |  |  |  |  |
| 0 | Vitvi11g04002\_t001 |  |  |  |  |  |  |  |  |  |  |  |  |
| 0 | Vitvi11g00002\_t001 |  |  |  |  |  |  |  |  |  |  |  |  |
| 0 | Vitvi11g00004\_t001 |  |  |  |  |  |  |  |  |  |  |  |  |
| 0 | Vitvi11g00005\_t004 |  |  |  |  |  |  |  |  |  |  |  |  |
| 0 | Vitvi11g00007\_t001 |  |  |  |  |  |  |  |  |  |  |  |  |
| 0 | Vitvi11g00008\_t001 |  |  |  |  |  |  |  |  |  |  |  |  |
| 0 | Vitvi11g00009\_t001 |  |  |  |  |  |  |  |  |  |  |  |  |
| 1 | Vitvi11g00010\_t001 |  | Vitvi09g00009\_t001 |  |  |  |  |  |  |  |  |  |  |
| 1 | Vitvi11g00011\_t001 |  | \| \| |  |  |  |  |  |  |  |  |  |  |
| 1 | Vitvi11g00012\_t001 |  | \| \| |  |  |  |  |  |  |  |  |  |  |
| 1 | Vitvi11g00013\_t001 |  | Vitvi09g00017\_t001 |  |  |  |  |  |  |  |  |  |  |
| 1 | Vitvi11g00014\_t001 |  | \| \| |  |  |  |  |  |  |  |  |  |  |
| 1 | Vitvi11g00015\_t001 |  | \| \| |  |  |  |  |  |  |  |  |  |  |
| 1 | Vitvi11g00016\_t001 |  | \| \| |  |  |  |  |  |  |  |  |  |  |
| 1 | Vitvi11g00017\_t001 |  | \| \| |  |  |  |  |  |  |  |  |  |  |
| 1 | Vitvi11g00018\_t001 |  | \| \| |  |  |  |  |  |  |  |  |  |  |
| 1 | Vitvi11g04003\_t001 |  | Vitvi09g00023\_t001 |  |  |  |  |  |  |  |  |  |  |
| 1 | Vitvi11g04004\_t001 |  | \| \| |  |  |  |  |  |  |  |  |  |  |
| 1 | Vitvi11g00020\_t001 |  | Vitvi09g00029\_t001 |  |  |  |  |  |  |  |  |  |  |
| 1 | Vitvi11g00021\_t001 |  | \| \| |  |  |  |  |  |  |  |  |  |  |
| 1 | Vitvi11g00022\_t001 |  | \| \| |  |  |  |  |  |  |  |  |  |  |
| 1 | Vitvi11g00023\_t001 |  | \| \| |  |  |  |  |  |  |  |  |  |  |
| 1 | Vitvi11g00024\_t001 |  | \| \| |  |  |  |  |  |  |  |  |  |  |
| 1 | Vitvi11g00025\_t001 |  | Vitvi09g00033\_t001 |  |  |  |  |  |  |  |  |  |  |
| 1 | Vitvi11g00026\_t001 |  | Vitvi09g00042\_t001 |  |  |  |  |  |  |  |  |  |  |
| 1 | Vitvi11g00027\_t001 |  | \| \| |  |  |  |  |  |  |  |  |  |  |
| 1 | Vitvi11g00028\_t001 |  | \| \| |  |  |  |  |  |  |  |  |  |  |
| 1 | Vitvi11g00030\_t001 |  | \| \| |  |  |  |  |  |  |  |  |  |  |
| 1 | Vitvi11g00031\_t001 |  | \| \| |  |  |  |  |  |  |  |  |  |  |
| 1 | Vitvi11g00032\_t001 |  | \| \| |  |  |  |  |  |  |  |  |  |  |
| 1 | Vitvi11g00033\_t001 |  | Vitvi09g00046\_t001 |  |  |  |  |  |  |  |  |  |  |
| 1 | Vitvi11g04005\_t001 |  | \| \| |  |  |  |  |  |  |  |  |  |  |
| 1 | Vitvi11g00034\_t001 |  | \| \| |  |  |  |  |  |  |  |  |  |  |
| 1 | Vitvi11g00035\_t001 |  | \| \| |  |  |  |  |  |  |  |  |  |  |
| 1 | Vitvi11g04006\_t001 |  | \| \| |  |  |  |  |  |  |  |  |  |  |
| 1 | Vitvi11g00036\_t002 |  | \| \| |  |  |  |  |  |  |  |  |  |  |
| 1 | Vitvi11g00037\_t001 |  | \| \| |  |  |  |  |  |  |  |  |  |  |
| 1 | Vitvi11g01324\_t001 |  | \| \| |  |  |  |  |  |  |  |  |  |  |
| 1 | Vitvi11g00038\_t001 |  | \| \| |  |  |  |  |  |  |  |  |  |  |
| 2 | Vitvi11g00039\_t001 |  | \| \| |  | Vitvi04g00193\_t001 |  |  |  |  |  |  |  |  |
| 2 | Vitvi11g04007\_t001 |  | \| \| |  | \| \| |  |  |  |  |  |  |  |  |
| 2 | Vitvi11g01325\_t001 |  | \| \| |  | \| \| |  |  |  |  |  |  |  |  |
| 2 | Vitvi11g00040\_t001 |  | \| \| |  | \| \| |  |  |  |  |  |  |  |  |
| 2 | Vitvi11g00041\_t002 |  | Vitvi09g00058\_t001 |  | \| \| |  |  |  |  |  |  |  |  |
| 2 | Vitvi11g01326\_t001 |  | \| \| |  | \| \| |  |  |  |  |  |  |  |  |
| 2 | Vitvi11g04008\_t001 |  | \| \| |  | \| \| |  |  |  |  |  |  |  |  |
| 2 | Vitvi11g00042\_t002 |  | \| \| |  | \| \| |  |  |  |  |  |  |  |  |
| 2 | Vitvi11g04009\_t001 |  | \| \| |  | \| \| |  |  |  |  |  |  |  |  |
| 2 | Vitvi11g00043\_t001 |  | \| \| |  | \| \| |  |  |  |  |  |  |  |  |
| 2 | Vitvi11g00044\_t002 |  | \| \| |  | \| \| |  |  |  |  |  |  |  |  |
| 2 | Vitvi11g00045\_t001 |  | \| \| |  | Vitvi04g00190\_t001 |  |  |  |  |  |  |  |  |
| 2 | Vitvi11g00046\_t001 |  | \| \| |  | \| \| |  |  |  |  |  |  |  |  |
| 2 | Vitvi11g00047\_t001 |  | \| \| |  | \| \| |  |  |  |  |  |  |  |  |
| 2 | Vitvi11g00048\_t001 |  | \| \| |  | \| \| |  |  |  |  |  |  |  |  |
| 2 | Vitvi11g00049\_t001 |  | Vitvi09g00063\_t001 |  | \| \| |  |  |  |  |  |  |  |  |
| 2 | Vitvi11g00050\_t001 |  | Vitvi09g00064\_t001 |  | \| \| |  |  |  |  |  |  |  |  |
| 2 | Vitvi11g00051\_t003 |  | \| \| |  | \| \| |  |  |  |  |  |  |  |  |
| 2 | Vitvi11g00052\_t001 |  | \| \| |  | \| \| |  |  |  |  |  |  |  |  |
| 2 | Vitvi11g00053\_t001 |  | Vitvi09g00065\_t001 |  | \| \| |  |  |  |  |  |  |  |  |
| 2 | Vitvi11g04010\_t001 |  | \| \| |  | \| \| |  |  |  |  |  |  |  |  |
| 2 | Vitvi11g04011\_t001 |  | \| \| |  | \| \| |  |  |  |  |  |  |  |  |
| 2 | Vitvi11g00055\_t001 |  | \| \| |  | \| \| |  |  |  |  |  |  |  |  |
| 2 | Vitvi11g00056\_t001 |  | \| \| |  | \| \| |  |  |  |  |  |  |  |  |
| 2 | Vitvi11g00057\_t001 |  | \| \| |  | \| \| |  |  |  |  |  |  |  |  |
| 2 | Vitvi11g00058\_t001 |  | Vitvi09g00069\_t001 |  | \| \| |  |  |  |  |  |  |  |  |
| 2 | Vitvi11g01327\_t001 |  | Vitvi09g01501\_t001 |  | \| \| |  |  |  |  |  |  |  |  |
| 2 | Vitvi11g00060\_t001 |  | \| \| |  | \| \| |  |  |  |  |  |  |  |  |
| 2 | Vitvi11g00061\_t001 |  | Vitvi09g00071\_t001 |  | \| \| |  |  |  |  |  |  |  |  |
| 2 | Vitvi11g00062\_t001 |  | \| \| |  | \| \| |  |  |  |  |  |  |  |  |
| 2 | Vitvi11g04012\_t001 |  | \| \| |  | \| \| |  |  |  |  |  |  |  |  |
| 2 | Vitvi11g00063\_t002 |  | \| \| |  | \| \| |  |  |  |  |  |  |  |  |
| 2 | Vitvi11g04013\_t001 |  | Vitvi09g04018\_t001 |  | \| \| |  |  |  |  |  |  |  |  |
| 2 | Vitvi11g00065\_t001 |  | \| \| |  | \| \| |  |  |  |  |  |  |  |  |
| 2 | Vitvi11g01328\_t001 |  | \| \| |  | \| \| |  |  |  |  |  |  |  |  |
| 2 | Vitvi11g01329\_t001 |  | \| \| |  | \| \| |  |  |  |  |  |  |  |  |
| 2 | Vitvi11g01330\_t001 |  | \| \| |  | \| \| |  |  |  |  |  |  |  |  |
| 2 | Vitvi11g01331\_t001 |  | Vitvi09g00077\_t001 |  | Vitvi04g00182\_t001 |  |  |  |  |  |  |  |  |
| 2 | Vitvi11g00066\_t001 |  | Vitvi09g00078\_t001 |  | \| \| |  |  |  |  |  |  |  |  |
| 2 | Vitvi11g00067\_t001 |  | Vitvi09g00079\_t001 |  | \| \| |  |  |  |  |  |  |  |  |
| 2 | Vitvi11g00068\_t001 |  | \| \| |  | Vitvi04g01813\_t001 |  |  |  |  |  |  |  |  |
| 2 | Vitvi11g00069\_t001 |  | \| \| |  | \| \| |  |  |  |  |  |  |  |  |
| 2 | Vitvi11g04014\_t001 |  | \| \| |  | \| \| |  |  |  |  |  |  |  |  |
| 2 | Vitvi11g00071\_t001 |  | Vitvi09g00081\_t001 |  | \| \| |  |  |  |  |  |  |  |  |
| 2 | Vitvi11g00072\_t001 |  | Vitvi09g00082\_t001 |  | \| \| |  |  |  |  |  |  |  |  |
| 2 | Vitvi11g01334\_t001 |  | Vitvi09g00086\_t001 |  | \| \| |  |  |  |  |  |  |  |  |
| 2 | Vitvi11g01335\_t001 |  | \| \| |  | \| \| |  |  |  |  |  |  |  |  |
| 2 | Vitvi11g04015\_t001 |  | \| \| |  | \| \| |  |  |  |  |  |  |  |  |
| 2 | Vitvi11g01336\_t001 |  | Vitvi09g00088\_t001 |  | Vitvi04g00179\_t001 |  |  |  |  |  |  |  |  |
| 2 | Vitvi11g00074\_t001 |  | \| \| |  | \| \| |  |  |  |  |  |  |  |  |
| 2 | Vitvi11g01337\_t001 |  | \| \| |  | \| \| |  |  |  |  |  |  |  |  |
| 2 | Vitvi11g01338\_t001 |  | \| \| |  | \| \| |  |  |  |  |  |  |  |  |
| 2 | Vitvi11g01339\_t001 |  | \| \| |  | \| \| |  |  |  |  |  |  |  |  |
| 2 | Vitvi11g00075\_t001 |  | \| \| |  | \| \| |  |  |  |  |  |  |  |  |
| 2 | Vitvi11g00076\_t001 |  | \| \| |  | \| \| |  |  |  |  |  |  |  |  |
| 2 | Vitvi11g04016\_t001 |  | \| \| |  | \| \| |  |  |  |  |  |  |  |  |
| 2 | Vitvi11g00077\_t001 |  | \| \| |  | \| \| |  |  |  |  |  |  |  |  |
| 2 | Vitvi11g01340\_t001 |  | \| \| |  | \| \| |  |  |  |  |  |  |  |  |
| 2 | Vitvi11g00078\_t001 |  | \| \| |  | \| \| |  |  |  |  |  |  |  |  |
| 2 | Vitvi11g00079\_t001 |  | \| \| |  | \| \| |  |  |  |  |  |  |  |  |
| 2 | Vitvi11g00080\_t001 |  | \| \| |  | Vitvi04g00173\_t001 |  |  |  |  |  |  |  |  |
| 2 | Vitvi11g00081\_t001 |  | \| \| |  | \| \| |  |  |  |  |  |  |  |  |
| 2 | Vitvi11g04017\_t001 |  | \| \| |  | \| \| |  |  |  |  |  |  |  |  |
| 2 | Vitvi11g01341\_t001 |  | \| \| |  | \| \| |  |  |  |  |  |  |  |  |
| 2 | Vitvi11g00083\_t001 |  | \| \| |  | \| \| |  |  |  |  |  |  |  |  |
| 2 | Vitvi11g00084\_t001 |  | \| \| |  | \| \| |  |  |  |  |  |  |  |  |
| 2 | Vitvi11g00085\_t001 |  | \| \| |  | Vitvi04g00165\_t001 |  |  |  |  |  |  |  |  |
| 2 | Vitvi11g00086\_t001 |  | \| \| |  | \| \| |  |  |  |  |  |  |  |  |
| 2 | Vitvi11g00087\_t001 |  | \| \| |  | \| \| |  |  |  |  |  |  |  |  |
| 2 | Vitvi11g00088\_t001 |  | \| \| |  | \| \| |  |  |  |  |  |  |  |  |
| 2 | Vitvi11g04018\_t001 |  | \| \| |  | \| \| |  |  |  |  |  |  |  |  |
| 2 | Vitvi11g00089\_t001 |  | Vitvi09g01512\_t001 |  | \| \| |  |  |  |  |  |  |  |  |
| 2 | Vitvi11g00090\_t001 |  | \| \| |  | \| \| |  |  |  |  |  |  |  |  |
| 2 | Vitvi11g00091\_t002 |  | \| \| |  | \| \| |  |  |  |  |  |  |  |  |
| 2 | Vitvi11g00092\_t001 |  | Vitvi09g00107\_t001 |  | \| \| |  |  |  |  |  |  |  |  |
| 2 | Vitvi11g04019\_t001 |  | \| \| |  | \| \| |  |  |  |  |  |  |  |  |
| 2 | Vitvi11g00093\_t001 |  | \| \| |  | \| \| |  |  |  |  |  |  |  |  |
| 2 | Vitvi11g00094\_t002 |  | \| \| |  | \| \| |  |  |  |  |  |  |  |  |
| 2 | Vitvi11g00095\_t001 |  | \| \| |  | \| \| |  |  |  |  |  |  |  |  |
| 2 | Vitvi11g00096\_t001 |  | \| \| |  | Vitvi04g00159\_t001 |  |  |  |  |  |  |  |  |
| 2 | Vitvi11g00097\_t001 |  | Vitvi09g00110\_t001 |  | Vitvi04g00153\_t001 |  |  |  |  |  |  |  |  |
| 2 | Vitvi11g00098\_t001 |  | \| \| |  | \| \| |  |  |  |  |  |  |  |  |
| 2 | Vitvi11g00099\_t001 |  | Vitvi09g00113\_t001 |  | \| \| |  |  |  |  |  |  |  |  |
| 2 | Vitvi11g01342\_t001 |  | \| \| |  | \| \| |  |  |  |  |  |  |  |  |
| 2 | Vitvi11g01343\_t001 |  | \| \| |  | \| \| |  |  |  |  |  |  |  |  |
| 2 | Vitvi11g00101\_t001 |  | \| \| |  | \| \| |  |  |  |  |  |  |  |  |
| 2 | Vitvi11g04020\_t001 |  | \| \| |  | \| \| |  |  |  |  |  |  |  |  |
| 2 | Vitvi11g00102\_t001 |  | Vitvi09g00117\_t001 |  | \| \| |  |  |  |  |  |  |  |  |
| 2 | Vitvi11g00103\_t001 |  | Vitvi09g00119\_t001 |  | Vitvi04g00149\_t001 |  |  |  |  |  |  |  |  |
| 2 | Vitvi11g00104\_t001 |  | \| \| |  | \| \| |  |  |  |  |  |  |  |  |
| 2 | Vitvi11g01345\_t001 |  | \| \| |  | Vitvi04g01806\_t001 |  |  |  |  |  |  |  |  |
| 2 | Vitvi11g01346\_t001 |  | \| \| |  | Vitvi04g00147\_t001 |  |  |  |  |  |  |  |  |
| 2 | Vitvi11g01347\_t001 |  | \| \| |  | \| \| |  |  |  |  |  |  |  |  |
| 2 | Vitvi11g01348\_t001 |  | Vitvi09g01517\_t001 |  | \| \| |  |  |  |  |  |  |  |  |
| 2 | Vitvi11g00105\_t001 |  | \| \| |  | \| \| |  |  |  |  |  |  |  |  |
| 2 | Vitvi11g00106\_t001 |  | Vitvi09g00126\_t001 |  | Vitvi04g00145\_t001 |  |  |  |  |  |  |  |  |
| 2 | Vitvi11g00107\_t001 |  | Vitvi09g00128\_t001 |  | \| \| |  |  |  |  |  |  |  |  |
| 2 | Vitvi11g00108\_t001 |  | Vitvi09g00129\_t002 |  | \| \| |  |  |  |  |  |  |  |  |
| 2 | Vitvi11g04021\_t001 |  | \| \| |  | \| \| |  |  |  |  |  |  |  |  |
| 2 | Vitvi11g00109\_t001 |  | Vitvi09g00133\_t001 |  | \| \| |  |  |  |  |  |  |  |  |
| 2 | Vitvi11g00110\_t001 |  | \| \| |  | \| \| |  |  |  |  |  |  |  |  |
| 2 | Vitvi11g00113\_t001 |  | Vitvi09g01518\_t001 |  | Vitvi04g00140\_t001 |  |  |  |  |  |  |  |  |
| 1 | Vitvi11g00115\_t001 |  |  |  | \| \| |  |  |  |  |  |  |  |  |
| 1 | Vitvi11g04022\_t001 |  |  |  | \| \| |  |  |  |  |  |  |  |  |
| 1 | Vitvi11g04023\_t001 |  |  |  | \| \| |  |  |  |  |  |  |  |  |
| 1 | Vitvi11g04024\_t001 |  |  |  | \| \| |  |  |  |  |  |  |  |  |
| 1 | Vitvi11g01349\_t001 |  |  |  | \| \| |  |  |  |  |  |  |  |  |
| 1 | Vitvi11g04025\_t001 |  |  |  | \| \| |  |  |  |  |  |  |  |  |
| 1 | Vitvi11g00117\_t001 |  |  |  | \| \| |  |  |  |  |  |  |  |  |
| 1 | Vitvi11g01350\_t001 |  |  |  | \| \| |  |  |  |  |  |  |  |  |
| 1 | Vitvi11g01351\_t001 |  |  |  | \| \| |  |  |  |  |  |  |  |  |
| 1 | Vitvi11g04026\_t001 |  |  |  | Vitvi04g00139\_t001 |  |  |  |  |  |  |  |  |
| 1 | Vitvi11g00119\_t001 |  |  |  | \| \| |  |  |  |  |  |  |  |  |
| 1 | Vitvi11g01353\_t001 |  |  |  | \| \| |  |  |  |  |  |  |  |  |
| 1 | Vitvi11g00120\_t001 |  |  |  | \| \| |  |  |  |  |  |  |  |  |
| 1 | Vitvi11g01355\_t001 |  |  |  | \| \| |  |  |  |  |  |  |  |  |
| 1 | Vitvi11g04027\_t001 |  |  |  | \| \| |  |  |  |  |  |  |  |  |
| 1 | Vitvi11g04028\_t001 |  |  |  | \| \| |  |  |  |  |  |  |  |  |
| 1 | Vitvi11g04029\_t001 |  |  |  | \| \| |  |  |  |  |  |  |  |  |
| 1 | Vitvi11g04030\_t001 |  |  |  | \| \| |  |  |  |  |  |  |  |  |
| 1 | Vitvi11g00125\_t001 |  |  |  | \| \| |  |  |  |  |  |  |  |  |
| 1 | Vitvi11g04031\_t001 |  |  |  | \| \| |  |  |  |  |  |  |  |  |
| 1 | Vitvi11g04032\_t001 |  |  |  | \| \| |  |  |  |  |  |  |  |  |
| 1 | Vitvi11g01357\_t001 |  |  |  | \| \| |  |  |  |  |  |  |  |  |
| 1 | Vitvi11g04033\_t001 |  |  |  | \| \| |  |  |  |  |  |  |  |  |
| 1 | Vitvi11g04034\_t001 |  |  |  | \| \| |  |  |  |  |  |  |  |  |
| 1 | Vitvi11g01359\_t001 |  |  |  | \| \| |  |  |  |  |  |  |  |  |
| 1 | Vitvi11g04035\_t001 |  |  |  | \| \| |  |  |  |  |  |  |  |  |
| 1 | Vitvi11g04036\_t001 |  |  |  | \| \| |  |  |  |  |  |  |  |  |
| 1 | Vitvi11g01360\_t001 |  |  |  | Vitvi04g04045\_t001 |  |  |  |  |  |  |  |  |
| 1 | Vitvi11g01361\_t001 |  |  |  | \| \| |  |  |  |  |  |  |  |  |
| 1 | Vitvi11g04037\_t001 |  |  |  | \| \| |  |  |  |  |  |  |  |  |
| 1 | Vitvi11g01362\_t001 |  |  |  | \| \| |  |  |  |  |  |  |  |  |
| 1 | Vitvi11g01363\_t001 |  |  |  | \| \| |  |  |  |  |  |  |  |  |
| 1 | Vitvi11g00127\_t002 |  |  |  | Vitvi04g00132\_t002 |  |  |  |  |  |  |  |  |
| 1 | Vitvi11g00128\_t001 |  |  |  | \| \| |  |  |  |  |  |  |  |  |
| 2 | Vitvi11g00129\_t001 |  | Vitvi09g00162\_t001 |  | \| \| |  |  |  |  |  |  |  |  |
| 2 | Vitvi11g00130\_t001 |  | \| \| |  | \| \| |  |  |  |  |  |  |  |  |
| 2 | Vitvi11g00131\_t001 |  | \| \| |  | \| \| |  |  |  |  |  |  |  |  |
| 2 | Vitvi11g00132\_t001 |  | Vitvi09g00159\_t001 |  | \| \| |  |  |  |  |  |  |  |  |
| 2 | Vitvi11g00133\_t001 |  | \| \| |  | \| \| |  |  |  |  |  |  |  |  |
| 2 | Vitvi11g00134\_t002 |  | Vitvi09g01525\_t002 |  | \| \| |  |  |  |  |  |  |  |  |
| 2 | Vitvi11g00135\_t001 |  | \| \| |  | \| \| |  |  |  |  |  |  |  |  |
| 2 | Vitvi11g00136\_t002 |  | \| \| |  | \| \| |  |  |  |  |  |  |  |  |
| 2 | Vitvi11g00137\_t002 |  | Vitvi09g00156\_t002 |  | Vitvi04g00127\_t001 |  |  |  |  |  |  |  |  |
| 2 | Vitvi11g00138\_t001 |  | \| \| |  | \| \| |  |  |  |  |  |  |  |  |
| 2 | Vitvi11g00139\_t001 |  | \| \| |  | Vitvi04g00124\_t001 |  |  |  |  |  |  |  |  |
| 2 | Vitvi11g00140\_t001 |  | \| \| |  | Vitvi04g00121\_t001 |  |  |  |  |  |  |  |  |
| 2 | Vitvi11g00141\_t001 |  | \| \| |  | \| \| |  |  |  |  |  |  |  |  |
| 2 | Vitvi11g00142\_t001 |  | \| \| |  | Vitvi04g00118\_t001 |  |  |  |  |  |  |  |  |
| 1 | Vitvi11g00143\_t001 |  | \| \| |  |  |  |  |  |  |  |  |  |  |
| 1 | Vitvi11g04038\_t001 |  | \| \| |  |  |  |  |  |  |  |  |  |  |
| 1 | Vitvi11g04039\_t001 |  | \| \| |  |  |  |  |  |  |  |  |  |  |
| 1 | Vitvi11g04040\_t001 |  | \| \| |  |  |  |  |  |  |  |  |  |  |
| 1 | Vitvi11g04041\_t001 |  | \| \| |  |  |  |  |  |  |  |  |  |  |
| 1 | Vitvi11g00146\_t001 |  | \| \| |  |  |  |  |  |  |  |  |  |  |
| 1 | Vitvi11g00147\_t001 |  | Vitvi09g00151\_t002 |  |  |  |  |  |  |  |  |  |  |
| 1 | Vitvi11g00148\_t001 |  | \| \| |  |  |  |  |  |  |  |  |  |  |
| 1 | Vitvi11g00149\_t001 |  | Vitvi09g04036\_t001 |  |  |  |  |  |  |  |  |  |  |
| 1 | Vitvi11g00150\_t001 |  | Vitvi09g00150\_t005 |  |  |  |  |  |  |  |  |  |  |
| 1 | Vitvi11g00151\_t001 |  | \| \| |  |  |  |  |  |  |  |  |  |  |
| 2 | Vitvi11g00152\_t001 |  | Vitvi09g00149\_t001 |  | Vitvi04g04031\_t001 |  |  |  |  |  |  |  |  |
| 2 | Vitvi11g00153\_t001 |  | \| \| |  | \| \| |  |  |  |  |  |  |  |  |
| 2 | Vitvi11g00154\_t001 |  | Vitvi09g01521\_t001 |  | Vitvi04g01786\_t001 |  |  |  |  |  |  |  |  |
| 2 | Vitvi11g00155\_t001 |  | Vitvi09g00148\_t001 |  | \| \| |  |  |  |  |  |  |  |  |
| 2 | Vitvi11g01366\_t001 |  | Vitvi09g04034\_t001 |  | \| \| |  |  |  |  |  |  |  |  |
| 2 | Vitvi11g00156\_t001 |  | Vitvi09g00144\_t001 |  | Vitvi04g00088\_t001 |  |  |  |  |  |  |  |  |
| 2 | Vitvi11g00157\_t002 |  | \| \| |  | \| \| |  |  |  |  |  |  |  |  |
| 2 | Vitvi11g00158\_t001 |  | \| \| |  | \| \| |  |  |  |  |  |  |  |  |
| 2 | Vitvi11g00159\_t001 |  | \| \| |  | Vitvi04g00092\_t001 |  |  |  |  |  |  |  |  |
| 2 | Vitvi11g00160\_t001 |  | Vitvi09g00140\_t001 |  | \| \| |  |  |  |  |  |  |  |  |
| 1 | Vitvi11g04042\_t001 |  |  |  | \| \| |  |  |  |  |  |  |  |  |
| 1 | Vitvi11g01367\_t001 |  |  |  | \| \| |  |  |  |  |  |  |  |  |
| 1 | Vitvi11g00161\_t001 |  |  |  | \| \| |  |  |  |  |  |  |  |  |
| 1 | Vitvi11g00162\_t001 |  |  |  | \| \| |  |  |  |  |  |  |  |  |
| 1 | Vitvi11g00163\_t001 |  |  |  | \| \| |  |  |  |  |  |  |  |  |
| 1 | Vitvi11g00164\_t001 |  |  |  | \| \| |  |  |  |  |  |  |  |  |
| 1 | Vitvi11g00165\_t001 |  |  |  | \| \| |  |  |  |  |  |  |  |  |
| 1 | Vitvi11g01369\_t001 |  |  |  | Vitvi04g00099\_t001 |  |  |  |  |  |  |  |  |
| 1 | Vitvi11g01370\_t001 |  |  |  | \| \| |  |  |  |  |  |  |  |  |
| 1 | Vitvi11g00167\_t001 |  |  |  | \| \| |  |  |  |  |  |  |  |  |
| 1 | Vitvi11g01371\_t001 |  |  |  | \| \| |  |  |  |  |  |  |  |  |
| 1 | Vitvi11g04043\_t001 |  |  |  | \| \| |  |  |  |  |  |  |  |  |
| 1 | Vitvi11g00168\_t001 |  |  |  | Vitvi04g04036\_t001 |  |  |  |  |  |  |  |  |
| 1 | Vitvi11g00169\_t001 |  |  |  | \| \| |  |  |  |  |  |  |  |  |
| 1 | Vitvi11g01372\_t001 |  |  |  | Vitvi04g00107\_t001 |  |  |  |  |  |  |  |  |
| 1 | Vitvi11g00170\_t001 |  |  |  | \| \| |  |  |  |  |  |  |  |  |
| 1 | Vitvi11g04044\_t001 |  |  |  | \| \| |  |  |  |  |  |  |  |  |
| 2 | Vitvi11g01322\_t001 |  | Vitvi09g00184\_t001 |  | \| \| |  |  |  |  |  |  |  |  |
| 2 | Vitvi11g01373\_t001 |  | \| \| |  | \| \| |  |  |  |  |  |  |  |  |
| 2 | Vitvi11g00174\_t001 |  | \| \| |  | \| \| |  |  |  |  |  |  |  |  |
| 2 | Vitvi11g01374\_t001 |  | \| \| |  | \| \| |  |  |  |  |  |  |  |  |
| 2 | Vitvi11g00175\_t001 |  | \| \| |  | \| \| |  |  |  |  |  |  |  |  |
| 2 | Vitvi11g00177\_t001 |  | \| \| |  | \| \| |  |  |  |  |  |  |  |  |
| 2 | Vitvi11g00178\_t001 |  | \| \| |  | \| \| |  |  |  |  |  |  |  |  |
| 3 | Vitvi11g00180\_t001 |  | \| \| |  | Vitvi04g00111\_t001 |  | Vitvi04g00111\_t001 |  |  |  |  |  |  |
| 3 | Vitvi11g04045\_t001 |  | \| \| |  | \| \| |  | \| \| |  |  |  |  |  |  |
| 3 | Vitvi11g00182\_t001 |  | \| \| |  | \| \| |  | \| \| |  |  |  |  |  |  |
| 3 | Vitvi11g04046\_t001 |  | \| \| |  | \| \| |  | \| \| |  |  |  |  |  |  |
| 3 | Vitvi11g00184\_t001 |  | \| \| |  | \| \| |  | \| \| |  |  |  |  |  |  |
| 3 | Vitvi11g00185\_t001 |  | \| \| |  | \| \| |  | \| \| |  |  |  |  |  |  |
| 3 | Vitvi11g00186\_t001 |  | \| \| |  | Vitvi04g00112\_t001 |  | \| \| |  |  |  |  |  |  |
| 2 | Vitvi11g00187\_t001 |  | \| \| |  |  |  | \| \| |  |  |  |  |  |  |
| 2 | Vitvi11g04047\_t001 |  | \| \| |  |  |  | \| \| |  |  |  |  |  |  |
| 2 | Vitvi11g00188\_t001 |  | \| \| |  |  |  | \| \| |  |  |  |  |  |  |
| 2 | Vitvi11g04048\_t001 |  | \| \| |  |  |  | \| \| |  |  |  |  |  |  |
| 2 | Vitvi11g00189\_t001 |  | Vitvi09g00199\_t002 |  |  |  | \| \| |  |  |  |  |  |  |
| 2 | Vitvi11g00190\_t001 |  | \| \| |  |  |  | \| \| |  |  |  |  |  |  |
| 2 | Vitvi11g00191\_t001 |  | Vitvi09g00201\_t001 |  |  |  | \| \| |  |  |  |  |  |  |
| 2 | Vitvi11g01376\_t001 |  | \| \| |  |  |  | \| \| |  |  |  |  |  |  |
| 2 | Vitvi11g00192\_t001 |  | \| \| |  |  |  | \| \| |  |  |  |  |  |  |
| 2 | Vitvi11g00193\_t001 |  | \| \| |  |  |  | \| \| |  |  |  |  |  |  |
| 2 | Vitvi11g00194\_t001 |  | \| \| |  |  |  | \| \| |  |  |  |  |  |  |
| 2 | Vitvi11g00195\_t001 |  | \| \| |  |  |  | \| \| |  |  |  |  |  |  |
| 2 | Vitvi11g00196\_t001 |  | \| \| |  |  |  | \| \| |  |  |  |  |  |  |
| 2 | Vitvi11g00197\_t001 |  | \| \| |  |  |  | \| \| |  |  |  |  |  |  |
| 2 | Vitvi11g00198\_t001 |  | \| \| |  |  |  | \| \| |  |  |  |  |  |  |
| 2 | Vitvi11g00199\_t002 |  | Vitvi09g00203\_t001 |  |  |  | \| \| |  |  |  |  |  |  |
| 2 | Vitvi11g00200\_t001 |  | \| \| |  |  |  | \| \| |  |  |  |  |  |  |
| 2 | Vitvi11g01377\_t001 |  | \| \| |  |  |  | \| \| |  |  |  |  |  |  |
| 2 | Vitvi11g01378\_t001 |  | \| \| |  |  |  | \| \| |  |  |  |  |  |  |
| 2 | Vitvi11g00201\_t001 |  | \| \| |  |  |  | Vitvi04g00087\_t001 |  |  |  |  |  |  |
| 2 | Vitvi11g00202\_t001 |  | Vitvi09g01542\_t002 |  |  |  | Vitvi04g00071\_t001 |  |  |  |  |  |  |
| 2 | Vitvi11g00203\_t001 |  | \| \| |  |  |  | Vitvi04g00070\_t001 |  |  |  |  |  |  |
| 2 | Vitvi11g00204\_t001 |  | \| \| |  |  |  | \| \| |  |  |  |  |  |  |
| 2 | Vitvi11g04049\_t001 |  | \| \| |  |  |  | \| \| |  |  |  |  |  |  |
| 2 | Vitvi11g00205\_t001 |  | \| \| |  |  |  | \| \| |  |  |  |  |  |  |
| 2 | Vitvi11g00206\_t001 |  | \| \| |  |  |  | Vitvi04g00069\_t001 |  |  |  |  |  |  |
| 2 | Vitvi11g00207\_t001 |  | \| \| |  |  |  | \| \| |  |  |  |  |  |  |
| 2 | Vitvi11g00208\_t001 |  | \| \| |  |  |  | \| \| |  |  |  |  |  |  |
| 2 | Vitvi11g00209\_t001 |  | \| \| |  |  |  | \| \| |  |  |  |  |  |  |
| 2 | Vitvi11g00210\_t001 |  | Vitvi09g00208\_t001 |  |  |  | Vitvi04g00068\_t001 |  |  |  |  |  |  |
| 2 | Vitvi11g00212\_t001 |  | \| \| |  |  |  | \| \| |  |  |  |  |  |  |
| 2 | Vitvi11g01379\_t001 |  | \| \| |  |  |  | \| \| |  |  |  |  |  |  |
| 2 | Vitvi11g00213\_t001 |  | \| \| |  |  |  | \| \| |  |  |  |  |  |  |
| 2 | Vitvi11g04050\_t001 |  | \| \| |  |  |  | \| \| |  |  |  |  |  |  |
| 2 | Vitvi11g00215\_t001 |  | \| \| |  |  |  | \| \| |  |  |  |  |  |  |
| 2 | Vitvi11g00217\_t002 |  | \| \| |  |  |  | \| \| |  |  |  |  |  |  |
| 2 | Vitvi11g00218\_t001 |  | \| \| |  |  |  | \| \| |  |  |  |  |  |  |
| 2 | Vitvi11g00219\_t001 |  | \| \| |  |  |  | \| \| |  |  |  |  |  |  |
| 2 | Vitvi11g00220\_t001 |  | \| \| |  |  |  | \| \| |  |  |  |  |  |  |
| 2 | Vitvi11g00221\_t001 |  | \| \| |  |  |  | \| \| |  |  |  |  |  |  |
| 2 | Vitvi11g00222\_t001 |  | \| \| |  |  |  | \| \| |  |  |  |  |  |  |
| 2 | Vitvi11g04051\_t001 |  | \| \| |  |  |  | \| \| |  |  |  |  |  |  |
| 2 | Vitvi11g04052\_t001 |  | \| \| |  |  |  | \| \| |  |  |  |  |  |  |
| 2 | Vitvi11g04053\_t001 |  | \| \| |  |  |  | \| \| |  |  |  |  |  |  |
| 2 | Vitvi11g04054\_t001 |  | \| \| |  |  |  | \| \| |  |  |  |  |  |  |
| 2 | Vitvi11g00223\_t001 |  | \| \| |  |  |  | \| \| |  |  |  |  |  |  |
| 2 | Vitvi11g00224\_t001 |  | \| \| |  |  |  | \| \| |  |  |  |  |  |  |
| 2 | Vitvi11g04055\_t001 |  | Vitvi09g04055\_t001 |  |  |  | \| \| |  |  |  |  |  |  |
| 2 | Vitvi11g04056\_t001 |  | \| \| |  |  |  | \| \| |  |  |  |  |  |  |
| 2 | Vitvi11g00226\_t001 |  | \| \| |  |  |  | \| \| |  |  |  |  |  |  |
| 2 | Vitvi11g01381\_t001 |  | Vitvi09g01550\_t006 |  |  |  | Vitvi04g01778\_t003 |  |  |  |  |  |  |
| 1 | Vitvi11g04057\_t001 |  |  |  |  |  | \| \| |  |  |  |  |  |  |
| 1 | Vitvi11g04058\_t001 |  |  |  |  |  | \| \| |  |  |  |  |  |  |
| 1 | Vitvi11g01385\_t001 |  |  |  |  |  | \| \| |  |  |  |  |  |  |
| 1 | Vitvi11g00227\_t001 |  |  |  |  |  | \| \| |  |  |  |  |  |  |
| 1 | Vitvi11g00228\_t001 |  |  |  |  |  | Vitvi04g00049\_t001 |  |  |  |  |  |  |
| 1 | Vitvi11g00229\_t001 |  |  |  |  |  | Vitvi04g00047\_t001 |  |  |  |  |  |  |
| 1 | Vitvi11g00231\_t001 |  |  |  |  |  | \| \| |  |  |  |  |  |  |
| 1 | Vitvi11g01386\_t001 |  |  |  |  |  | Vitvi04g00045\_t001 |  |  |  |  |  |  |
| 1 | Vitvi11g00233\_t001 |  |  |  |  |  | \| \| |  |  |  |  |  |  |
| 1 | Vitvi11g00234\_t001 |  |  |  |  |  | \| \| |  |  |  |  |  |  |
| 1 | Vitvi11g00235\_t001 |  |  |  |  |  | \| \| |  |  |  |  |  |  |
| 1 | Vitvi11g00237\_t001 |  |  |  |  |  | Vitvi04g00040\_t003 |  |  |  |  |  |  |
| 1 | Vitvi11g00238\_t001 |  |  |  |  |  | \| \| |  |  |  |  |  |  |
| 2 | Vitvi11g00240\_t001 |  | Vitvi09g00271\_t001 |  |  |  | \| \| |  |  |  |  |  |  |
| 2 | Vitvi11g00241\_t001 |  | \| \| |  |  |  | \| \| |  |  |  |  |  |  |
| 2 | Vitvi11g00242\_t001 |  | \| \| |  |  |  | \| \| |  |  |  |  |  |  |
| 2 | Vitvi11g00243\_t001 |  | \| \| |  |  |  | \| \| |  |  |  |  |  |  |
| 2 | Vitvi11g00244\_t001 |  | Vitvi09g01562\_t001 |  |  |  | \| \| |  |  |  |  |  |  |
| 2 | Vitvi11g00245\_t001 |  | \| \| |  |  |  | \| \| |  |  |  |  |  |  |
| 2 | Vitvi11g00247\_t001 |  | Vitvi09g00273\_t001 |  |  |  | Vitvi04g00038\_t001 |  |  |  |  |  |  |
| 2 | Vitvi11g00248\_t001 |  | \| \| |  |  |  | \| \| |  |  |  |  |  |  |
| 2 | Vitvi11g00249\_t001 |  | \| \| |  |  |  | \| \| |  |  |  |  |  |  |
| 2 | Vitvi11g00250\_t001 |  | \| \| |  |  |  | \| \| |  |  |  |  |  |  |
| 2 | Vitvi11g00251\_t001 |  | Vitvi09g00275\_t001 |  |  |  | Vitvi04g00036\_t001 |  |  |  |  |  |  |
| 2 | Vitvi11g04059\_t001 |  | \| \| |  |  |  | \| \| |  |  |  |  |  |  |
| 2 | Vitvi11g00252\_t001 |  | \| \| |  |  |  | \| \| |  |  |  |  |  |  |
| 2 | Vitvi11g00254\_t001 |  | \| \| |  |  |  | \| \| |  |  |  |  |  |  |
| 2 | Vitvi11g00255\_t001 |  | \| \| |  |  |  | \| \| |  |  |  |  |  |  |
| 2 | Vitvi11g00256\_t001 |  | \| \| |  |  |  | \| \| |  |  |  |  |  |  |
| 2 | Vitvi11g00257\_t001 |  | Vitvi09g00276\_t002 |  |  |  | \| \| |  |  |  |  |  |  |
| 2 | Vitvi11g00258\_t001 |  | Vitvi09g00277\_t001 |  |  |  | \| \| |  |  |  |  |  |  |
| 2 | Vitvi11g00259\_t001 |  | \| \| |  |  |  | \| \| |  |  |  |  |  |  |
| 2 | Vitvi11g04060\_t001 |  | \| \| |  |  |  | \| \| |  |  |  |  |  |  |
| 2 | Vitvi11g01390\_t001 |  | \| \| |  |  |  | \| \| |  |  |  |  |  |  |
| 2 | Vitvi11g00260\_t001 |  | Vitvi09g00279\_t001 |  |  |  | \| \| |  |  |  |  |  |  |
| 2 | Vitvi11g00261\_t001 |  | \| \| |  |  |  | Vitvi04g00021\_t001 |  |  |  |  |  |  |
| 2 | Vitvi11g00262\_t001 |  | \| \| |  |  |  | \| \| |  |  |  |  |  |  |
| 2 | Vitvi11g00263\_t001 |  | \| \| |  |  |  | \| \| |  |  |  |  |  |  |
| 2 | Vitvi11g00265\_t001 |  | \| \| |  |  |  | \| \| |  |  |  |  |  |  |
| 2 | Vitvi11g00266\_t001 |  | \| \| |  |  |  | \| \| |  |  |  |  |  |  |
| 2 | Vitvi11g00267\_t001 |  | \| \| |  |  |  | \| \| |  |  |  |  |  |  |
| 2 | Vitvi11g00268\_t001 |  | \| \| |  |  |  | \| \| |  |  |  |  |  |  |
| 2 | Vitvi11g00269\_t001 |  | \| \| |  |  |  | \| \| |  |  |  |  |  |  |
| 2 | Vitvi11g01392\_t001 |  | Vitvi09g00290\_t001 |  |  |  | Vitvi04g00012\_t001 |  |  |  |  |  |  |
| 2 | Vitvi11g00270\_t001 |  | Vitvi09g04087\_t001 |  |  |  | \| \| |  |  |  |  |  |  |
| 2 | Vitvi11g01393\_t001 |  | \| \| |  |  |  | \| \| |  |  |  |  |  |  |
| 2 | Vitvi11g00271\_t002 |  | \| \| |  |  |  | \| \| |  |  |  |  |  |  |
| 2 | Vitvi11g00272\_t001 |  | Vitvi09g00301\_t001 |  |  |  | Vitvi04g00009\_t001 |  |  |  |  |  |  |
| 2 | Vitvi11g00273\_t002 |  | \| \| |  |  |  | \| \| |  |  |  |  |  |  |
| 2 | Vitvi11g00274\_t001 |  | \| \| |  |  |  | \| \| |  |  |  |  |  |  |
| 2 | Vitvi11g00275\_t001 |  | Vitvi09g00307\_t001 |  |  |  | Vitvi04g00005\_t001 |  |  |  |  |  |  |
| 1 | Vitvi11g04061\_t001 |  |  |  |  |  | \| \| |  |  |  |  |  |  |
| 2 | Vitvi11g00276\_t001 |  | Vitvi04g00282\_t001 |  |  |  | Vitvi04g00001\_t001 |  |  |  |  |  |  |
| 1 | Vitvi11g00277\_t001 |  | \| \| |  |  |  |  |  |  |  |  |  |  |
| 1 | Vitvi11g00278\_t001 |  | \| \| |  |  |  |  |  |  |  |  |  |  |
| 1 | Vitvi11g00279\_t001 |  | \| \| |  |  |  |  |  |  |  |  |  |  |
| 1 | Vitvi11g00280\_t001 |  | \| \| |  |  |  |  |  |  |  |  |  |  |
| 1 | Vitvi11g00282\_t001 |  | \| \| |  |  |  |  |  |  |  |  |  |  |
| 1 | Vitvi11g00283\_t001 |  | Vitvi04g00294\_t002 |  |  |  |  |  |  |  |  |  |  |
| 1 | Vitvi11g00284\_t001 |  | \| \| |  |  |  |  |  |  |  |  |  |  |
| 1 | Vitvi11g04062\_t001 |  | \| \| |  |  |  |  |  |  |  |  |  |  |
| 2 | Vitvi11g00285\_t001 |  | Vitvi04g00300\_t001 |  | Vitvi09g00323\_t001 |  |  |  |  |  |  |  |  |
| 2 | Vitvi11g00286\_t001 |  | \| \| |  | \| \| |  |  |  |  |  |  |  |  |
| 2 | Vitvi11g01394\_t001 |  | Vitvi04g01841\_t001 |  | \| \| |  |  |  |  |  |  |  |  |
| 2 | Vitvi11g00287\_t001 |  | \| \| |  | \| \| |  |  |  |  |  |  |  |  |
| 2 | Vitvi11g00288\_t001 |  | \| \| |  | \| \| |  |  |  |  |  |  |  |  |
| 2 | Vitvi11g00290\_t001 |  | \| \| |  | \| \| |  |  |  |  |  |  |  |  |
| 2 | Vitvi11g00292\_t001 |  | \| \| |  | \| \| |  |  |  |  |  |  |  |  |
| 2 | Vitvi11g00293\_t001 |  | Vitvi04g04067\_t001 |  | \| \| |  |  |  |  |  |  |  |  |
| 2 | Vitvi11g00294\_t001 |  | \| \| |  | \| \| |  |  |  |  |  |  |  |  |
| 2 | Vitvi11g04063\_t001 |  | \| \| |  | \| \| |  |  |  |  |  |  |  |  |
| 2 | Vitvi11g00295\_t001 |  | Vitvi04g01849\_t001 |  | \| \| |  |  |  |  |  |  |  |  |
| 2 | Vitvi11g00296\_t001 |  | \| \| |  | \| \| |  |  |  |  |  |  |  |  |
| 2 | Vitvi11g04064\_t001 |  | \| \| |  | \| \| |  |  |  |  |  |  |  |  |
| 2 | Vitvi11g01396\_t001 |  | \| \| |  | \| \| |  |  |  |  |  |  |  |  |
| 2 | Vitvi11g01397\_t001 |  | \| \| |  | \| \| |  |  |  |  |  |  |  |  |
| 2 | Vitvi11g01398\_t001 |  | \| \| |  | \| \| |  |  |  |  |  |  |  |  |
| 2 | Vitvi11g00297\_t001 |  | Vitvi04g00309\_t001 |  | \| \| |  |  |  |  |  |  |  |  |
| 2 | Vitvi11g01399\_t001 |  | \| \| |  | \| \| |  |  |  |  |  |  |  |  |
| 2 | Vitvi11g00298\_t001 |  | \| \| |  | \| \| |  |  |  |  |  |  |  |  |
| 2 | Vitvi11g00299\_t001 |  | \| \| |  | \| \| |  |  |  |  |  |  |  |  |
| 2 | Vitvi11g00300\_t001 |  | \| \| |  | \| \| |  |  |  |  |  |  |  |  |
| 2 | Vitvi11g00301\_t001 |  | \| \| |  | Vitvi09g00336\_t002 |  |  |  |  |  |  |  |  |
| 2 | Vitvi11g00302\_t001 |  | \| \| |  | \| \| |  |  |  |  |  |  |  |  |
| 2 | Vitvi11g00303\_t001 |  | \| \| |  | Vitvi09g00339\_t001 |  |  |  |  |  |  |  |  |
| 2 | Vitvi11g00304\_t001 |  | Vitvi04g00312\_t001 |  | \| \| |  |  |  |  |  |  |  |  |
| 2 | Vitvi11g00305\_t001 |  | \| \| |  | \| \| |  |  |  |  |  |  |  |  |
| 2 | Vitvi11g00306\_t001 |  | \| \| |  | Vitvi09g00340\_t001 |  |  |  |  |  |  |  |  |
| 2 | Vitvi11g00307\_t001 |  | \| \| |  | \| \| |  |  |  |  |  |  |  |  |
| 2 | Vitvi11g00308\_t001 |  | \| \| |  | \| \| |  |  |  |  |  |  |  |  |
| 2 | Vitvi11g00309\_t001 |  | \| \| |  | \| \| |  |  |  |  |  |  |  |  |
| 2 | Vitvi11g00310\_t001 |  | \| \| |  | \| \| |  |  |  |  |  |  |  |  |
| 2 | Vitvi11g04065\_t001 |  | \| \| |  | \| \| |  |  |  |  |  |  |  |  |
| 2 | Vitvi11g01400\_t001 |  | \| \| |  | \| \| |  |  |  |  |  |  |  |  |
| 2 | Vitvi11g04066\_t001 |  | \| \| |  | \| \| |  |  |  |  |  |  |  |  |
| 2 | Vitvi11g00312\_t001 |  | \| \| |  | \| \| |  |  |  |  |  |  |  |  |
| 2 | Vitvi11g00314\_t001 |  | Vitvi04g00320\_t001 |  | \| \| |  |  |  |  |  |  |  |  |
| 2 | Vitvi11g00315\_t001 |  | \| \| |  | \| \| |  |  |  |  |  |  |  |  |
| 2 | Vitvi11g00316\_t001 |  | \| \| |  | \| \| |  |  |  |  |  |  |  |  |
| 2 | Vitvi11g00317\_t001 |  | \| \| |  | \| \| |  |  |  |  |  |  |  |  |
| 2 | Vitvi11g00318\_t001 |  | Vitvi04g00328\_t001 |  | \| \| |  |  |  |  |  |  |  |  |
| 2 | Vitvi11g00319\_t001 |  | \| \| |  | \| \| |  |  |  |  |  |  |  |  |
| 2 | Vitvi11g00320\_t001 |  | Vitvi04g01855\_t001 |  | \| \| |  |  |  |  |  |  |  |  |
| 2 | Vitvi11g04067\_t001 |  | \| \| |  | \| \| |  |  |  |  |  |  |  |  |
| 2 | Vitvi11g00321\_t001 |  | \| \| |  | \| \| |  |  |  |  |  |  |  |  |
| 2 | Vitvi11g00322\_t001 |  | \| \| |  | Vitvi09g00355\_t001 |  |  |  |  |  |  |  |  |
| 2 | Vitvi11g00323\_t001 |  | \| \| |  | \| \| |  |  |  |  |  |  |  |  |
| 2 | Vitvi11g04068\_t001 |  | \| \| |  | \| \| |  |  |  |  |  |  |  |  |
| 2 | Vitvi11g00326\_t001 |  | \| \| |  | \| \| |  |  |  |  |  |  |  |  |
| 2 | Vitvi11g04069\_t001 |  | \| \| |  | \| \| |  |  |  |  |  |  |  |  |
| 2 | Vitvi11g00327\_t001 |  | \| \| |  | \| \| |  |  |  |  |  |  |  |  |
| 2 | Vitvi11g00328\_t001 |  | Vitvi04g00336\_t001 |  | \| \| |  |  |  |  |  |  |  |  |
| 2 | Vitvi11g00329\_t001 |  | Vitvi04g00337\_t001 |  | \| \| |  |  |  |  |  |  |  |  |
| 2 | Vitvi11g00330\_t001 |  | \| \| |  | \| \| |  |  |  |  |  |  |  |  |
| 2 | Vitvi11g00331\_t001 |  | \| \| |  | \| \| |  |  |  |  |  |  |  |  |
| 2 | Vitvi11g00332\_t001 |  | \| \| |  | \| \| |  |  |  |  |  |  |  |  |
| 2 | Vitvi11g00333\_t001 |  | \| \| |  | \| \| |  |  |  |  |  |  |  |  |
| 2 | Vitvi11g00334\_t001 |  | \| \| |  | \| \| |  |  |  |  |  |  |  |  |
| 2 | Vitvi11g00335\_t001 |  | \| \| |  | \| \| |  |  |  |  |  |  |  |  |
| 2 | Vitvi11g00336\_t001 |  | \| \| |  | \| \| |  |  |  |  |  |  |  |  |
| 2 | Vitvi11g00337\_t001 |  | \| \| |  | \| \| |  |  |  |  |  |  |  |  |
| 2 | Vitvi11g01403\_t001 |  | \| \| |  | Vitvi09g00372\_t001 |  |  |  |  |  |  |  |  |
| 2 | Vitvi11g00338\_t001 |  | Vitvi04g00340\_t001 |  | \| \| |  |  |  |  |  |  |  |  |
| 2 | Vitvi11g04070\_t001 |  | \| \| |  | \| \| |  |  |  |  |  |  |  |  |
| 2 | Vitvi11g00339\_t001 |  | \| \| |  | \| \| |  |  |  |  |  |  |  |  |
| 2 | Vitvi11g00340\_t001 |  | Vitvi04g01873\_t006 |  | \| \| |  |  |  |  |  |  |  |  |
| 2 | Vitvi11g00341\_t001 |  | \| \| |  | Vitvi09g00373\_t001 |  |  |  |  |  |  |  |  |
| 2 | Vitvi11g00342\_t001 |  | \| \| |  | \| \| |  |  |  |  |  |  |  |  |
| 2 | Vitvi11g00343\_t001 |  | \| \| |  | Vitvi09g00378\_t001 |  |  |  |  |  |  |  |  |
| 2 | Vitvi11g01404\_t001 |  | \| \| |  | \| \| |  |  |  |  |  |  |  |  |
| 2 | Vitvi11g00344\_t001 |  | \| \| |  | \| \| |  |  |  |  |  |  |  |  |
| 2 | Vitvi11g01405\_t001 |  | \| \| |  | \| \| |  |  |  |  |  |  |  |  |
| 2 | Vitvi11g00346\_t001 |  | \| \| |  | Vitvi09g00385\_t001 |  |  |  |  |  |  |  |  |
| 2 | Vitvi11g01406\_t001 |  | \| \| |  | \| \| |  |  |  |  |  |  |  |  |
| 2 | Vitvi11g00347\_t003 |  | \| \| |  | Vitvi09g00387\_t001 |  |  |  |  |  |  |  |  |
| 2 | Vitvi11g04071\_t001 |  | \| \| |  | \| \| |  |  |  |  |  |  |  |  |
| 2 | Vitvi11g00348\_t001 |  | \| \| |  | Vitvi09g00388\_t001 |  |  |  |  |  |  |  |  |
| 2 | Vitvi11g04072\_t001 |  | \| \| |  | \| \| |  |  |  |  |  |  |  |  |
| 2 | Vitvi11g04073\_t001 |  | \| \| |  | \| \| |  |  |  |  |  |  |  |  |
| 2 | Vitvi11g00349\_t002 |  | \| \| |  | \| \| |  |  |  |  |  |  |  |  |
| 2 | Vitvi11g01409\_t001 |  | Vitvi04g04088\_t001 |  | Vitvi09g04113\_t001 |  |  |  |  |  |  |  |  |
| 2 | Vitvi11g00350\_t001 |  | \| \| |  | \| \| |  |  |  |  |  |  |  |  |
| 2 | Vitvi11g00351\_t001 |  | \| \| |  | \| \| |  |  |  |  |  |  |  |  |
| 2 | Vitvi11g00352\_t001 |  | \| \| |  | \| \| |  |  |  |  |  |  |  |  |
| 2 | Vitvi11g00353\_t001 |  | \| \| |  | \| \| |  |  |  |  |  |  |  |  |
| 2 | Vitvi11g01410\_t001 |  | \| \| |  | \| \| |  |  |  |  |  |  |  |  |
| 2 | Vitvi11g00354\_t002 |  | \| \| |  | Vitvi09g00393\_t001 |  |  |  |  |  |  |  |  |
| 2 | Vitvi11g01411\_t001 |  | \| \| |  | \| \| |  |  |  |  |  |  |  |  |
| 2 | Vitvi11g00355\_t001 |  | \| \| |  | Vitvi09g00399\_t001 |  |  |  |  |  |  |  |  |
| 2 | Vitvi11g00356\_t001 |  | \| \| |  | \| \| |  |  |  |  |  |  |  |  |
| 2 | Vitvi11g00357\_t001 |  | \| \| |  | \| \| |  |  |  |  |  |  |  |  |
| 2 | Vitvi11g00359\_t001 |  | \| \| |  | Vitvi09g00401\_t003 |  |  |  |  |  |  |  |  |
| 2 | Vitvi11g04074\_t001 |  | \| \| |  | \| \| |  |  |  |  |  |  |  |  |
| 2 | Vitvi11g00360\_t001 |  | \| \| |  | \| \| |  |  |  |  |  |  |  |  |
| 2 | Vitvi11g04075\_t001 |  | \| \| |  | \| \| |  |  |  |  |  |  |  |  |
| 2 | Vitvi11g04076\_t001 |  | \| \| |  | \| \| |  |  |  |  |  |  |  |  |
| 2 | Vitvi11g00362\_t001 |  | \| \| |  | \| \| |  |  |  |  |  |  |  |  |
| 3 | Vitvi11g00363\_t001 |  | \| \| |  | Vitvi09g00404\_t003 |  | Vitvi04g00384\_t001 |  |  |  |  |  |  |
| 3 | Vitvi11g04077\_t001 |  | \| \| |  | \| \| |  | \| \| |  |  |  |  |  |  |
| 3 | Vitvi11g00364\_t001 |  | \| \| |  | Vitvi09g00405\_t001 |  | \| \| |  |  |  |  |  |  |
| 2 | Vitvi11g00365\_t001 |  | \| \| |  |  |  | \| \| |  |  |  |  |  |  |
| 2 | Vitvi11g00366\_t001 |  | \| \| |  |  |  | \| \| |  |  |  |  |  |  |
| 2 | Vitvi11g04078\_t001 |  | \| \| |  |  |  | \| \| |  |  |  |  |  |  |
| 2 | Vitvi11g00367\_t001 |  | Vitvi04g00381\_t002 |  |  |  | \| \| |  |  |  |  |  |  |
| 1 | Vitvi11g00368\_t001 |  |  |  |  |  | \| \| |  |  |  |  |  |  |
| 1 | Vitvi11g00370\_t001 |  |  |  |  |  | \| \| |  |  |  |  |  |  |
| 1 | Vitvi11g01413\_t001 |  |  |  |  |  | \| \| |  |  |  |  |  |  |
| 1 | Vitvi11g00371\_t001 |  |  |  |  |  | \| \| |  |  |  |  |  |  |
| 1 | Vitvi11g00372\_t001 |  |  |  |  |  | \| \| |  |  |  |  |  |  |
| 1 | Vitvi11g04079\_t001 |  |  |  |  |  | \| \| |  |  |  |  |  |  |
| 1 | Vitvi11g04080\_t001 |  |  |  |  |  | \| \| |  |  |  |  |  |  |
| 1 | Vitvi11g00373\_t001 |  |  |  |  |  | \| \| |  |  |  |  |  |  |
| 1 | Vitvi11g00375\_t001 |  |  |  |  |  | \| \| |  |  |  |  |  |  |
| 1 | Vitvi11g01414\_t001 |  |  |  |  |  | \| \| |  |  |  |  |  |  |
| 1 | Vitvi11g04081\_t001 |  |  |  |  |  | \| \| |  |  |  |  |  |  |
| 1 | Vitvi11g01415\_t001 |  |  |  |  |  | \| \| |  |  |  |  |  |  |
| 1 | Vitvi11g00376\_t001 |  |  |  |  |  | \| \| |  |  |  |  |  |  |
| 1 | Vitvi11g00378\_t001 |  |  |  |  |  | \| \| |  |  |  |  |  |  |
| 1 | Vitvi11g00380\_t001 |  |  |  |  |  | \| \| |  |  |  |  |  |  |
| 1 | Vitvi11g00381\_t001 |  |  |  |  |  | Vitvi04g00378\_t001 |  |  |  |  |  |  |
| 1 | Vitvi11g04082\_t001 |  |  |  |  |  | \| \| |  |  |  |  |  |  |
| 1 | Vitvi11g00383\_t001 |  |  |  |  |  | \| \| |  |  |  |  |  |  |
| 1 | Vitvi11g04083\_t001 |  |  |  |  |  | \| \| |  |  |  |  |  |  |
| 1 | Vitvi11g00385\_t001 |  |  |  |  |  | Vitvi04g01874\_t001 |  |  |  |  |  |  |
| 1 | Vitvi11g00386\_t001 |  |  |  |  |  | \| \| |  |  |  |  |  |  |
| 1 | Vitvi11g04084\_t001 |  |  |  |  |  | \| \| |  |  |  |  |  |  |
| 1 | Vitvi11g04085\_t001 |  |  |  |  |  | \| \| |  |  |  |  |  |  |
| 1 | Vitvi11g00387\_t001 |  |  |  |  |  | \| \| |  |  |  |  |  |  |
| 1 | Vitvi11g04086\_t001 |  |  |  |  |  | \| \| |  |  |  |  |  |  |
| 1 | Vitvi11g04087\_t001 |  |  |  |  |  | \| \| |  |  |  |  |  |  |
| 1 | Vitvi11g00389\_t001 |  |  |  |  |  | \| \| |  |  |  |  |  |  |
| 1 | Vitvi11g00390\_t001 |  |  |  |  |  | Vitvi04g00372\_t001 |  |  |  |  |  |  |
| 1 | Vitvi11g01418\_t001 |  |  |  |  |  | \| \| |  |  |  |  |  |  |
| 1 | Vitvi11g04088\_t001 |  |  |  |  |  | \| \| |  |  |  |  |  |  |
| 1 | Vitvi11g01419\_t001 |  |  |  |  |  | \| \| |  |  |  |  |  |  |
| 1 | Vitvi11g00393\_t001 |  |  |  |  |  | \| \| |  |  |  |  |  |  |
| 1 | Vitvi11g00394\_t001 |  |  |  |  |  | \| \| |  |  |  |  |  |  |
| 1 | Vitvi11g00395\_t002 |  |  |  |  |  | Vitvi04g00371\_t001 |  |  |  |  |  |  |
| 1 | Vitvi11g00396\_t001 |  |  |  |  |  | \| \| |  |  |  |  |  |  |
| 1 | Vitvi11g00397\_t001 |  |  |  |  |  | \| \| |  |  |  |  |  |  |
| 3 | Vitvi11g00398\_t001 |  | Vitvi04g00421\_t001 |  | Vitvi09g00462\_t001 |  | Vitvi04g00365\_t001 |  |  |  |  |  |  |
| 2 | Vitvi11g00399\_t001 |  | Vitvi04g00431\_t003 |  | \| \| |  |  |  |  |  |  |  |  |
| 2 | Vitvi11g00400\_t001 |  | \| \| |  | \| \| |  |  |  |  |  |  |  |  |
| 2 | Vitvi11g00401\_t001 |  | \| \| |  | \| \| |  |  |  |  |  |  |  |  |
| 2 | Vitvi11g00402\_t001 |  | \| \| |  | \| \| |  |  |  |  |  |  |  |  |
| 2 | Vitvi11g00403\_t001 |  | \| \| |  | \| \| |  |  |  |  |  |  |  |  |
| 2 | Vitvi11g00405\_t001 |  | \| \| |  | \| \| |  |  |  |  |  |  |  |  |
| 2 | Vitvi11g00406\_t001 |  | \| \| |  | \| \| |  |  |  |  |  |  |  |  |
| 2 | Vitvi11g04089\_t001 |  | \| \| |  | \| \| |  |  |  |  |  |  |  |  |
| 2 | Vitvi11g00408\_t001 |  | \| \| |  | \| \| |  |  |  |  |  |  |  |  |
| 2 | Vitvi11g04090\_t001 |  | \| \| |  | \| \| |  |  |  |  |  |  |  |  |
| 2 | Vitvi11g00409\_t001 |  | \| \| |  | \| \| |  |  |  |  |  |  |  |  |
| 2 | Vitvi11g00410\_t001 |  | \| \| |  | \| \| |  |  |  |  |  |  |  |  |
| 2 | Vitvi11g00411\_t001 |  | \| \| |  | \| \| |  |  |  |  |  |  |  |  |
| 2 | Vitvi11g00412\_t001 |  | \| \| |  | \| \| |  |  |  |  |  |  |  |  |
| 2 | Vitvi11g00413\_t001 |  | \| \| |  | \| \| |  |  |  |  |  |  |  |  |
| 2 | Vitvi11g01421\_t001 |  | \| \| |  | \| \| |  |  |  |  |  |  |  |  |
| 2 | Vitvi11g00414\_t003 |  | \| \| |  | \| \| |  |  |  |  |  |  |  |  |
| 2 | Vitvi11g00415\_t001 |  | \| \| |  | \| \| |  |  |  |  |  |  |  |  |
| 2 | Vitvi11g00416\_t001 |  | \| \| |  | Vitvi09g00496\_t001 |  |  |  |  |  |  |  |  |
| 2 | Vitvi11g00417\_t001 |  | \| \| |  | \| \| |  |  |  |  |  |  |  |  |
| 2 | Vitvi11g04091\_t001 |  | \| \| |  | \| \| |  |  |  |  |  |  |  |  |
| 2 | Vitvi11g00418\_t001 |  | \| \| |  | \| \| |  |  |  |  |  |  |  |  |
| 2 | Vitvi11g00419\_t001 |  | Vitvi04g00439\_t001 |  | Vitvi09g00500\_t001 |  |  |  |  |  |  |  |  |
| 2 | Vitvi11g00420\_t001 |  | Vitvi04g00440\_t001 |  | \| \| |  |  |  |  |  |  |  |  |
| 2 | Vitvi11g01422\_t001 |  | \| \| |  | Vitvi09g01641\_t001 |  |  |  |  |  |  |  |  |
| 2 | Vitvi11g01423\_t001 |  | \| \| |  | \| \| |  |  |  |  |  |  |  |  |
| 2 | Vitvi11g04092\_t001 |  | \| \| |  | \| \| |  |  |  |  |  |  |  |  |
| 2 | Vitvi11g04093\_t001 |  | Vitvi04g04112\_t001 |  | Vitvi09g00513\_t001 |  |  |  |  |  |  |  |  |
| 2 | Vitvi11g04094\_t001 |  | \| \| |  | \| \| |  |  |  |  |  |  |  |  |
| 2 | Vitvi11g00424\_t001 |  | \| \| |  | Vitvi09g00516\_t001 |  |  |  |  |  |  |  |  |
| 2 | Vitvi11g00425\_t003 |  | Vitvi04g00444\_t001 |  | \| \| |  |  |  |  |  |  |  |  |
| 2 | Vitvi11g04095\_t001 |  | \| \| |  | \| \| |  |  |  |  |  |  |  |  |
| 2 | Vitvi11g00426\_t001 |  | \| \| |  | \| \| |  |  |  |  |  |  |  |  |
| 2 | Vitvi11g00427\_t001 |  | \| \| |  | \| \| |  |  |  |  |  |  |  |  |
| 2 | Vitvi11g00428\_t001 |  | Vitvi04g00454\_t001 |  | Vitvi09g00518\_t001 |  |  |  |  |  |  |  |  |
| 2 | Vitvi11g00429\_t001 |  | \| \| |  | \| \| |  |  |  |  |  |  |  |  |
| 2 | Vitvi11g00430\_t001 |  | \| \| |  | Vitvi09g00522\_t001 |  |  |  |  |  |  |  |  |
| 2 | Vitvi11g04096\_t001 |  | \| \| |  | \| \| |  |  |  |  |  |  |  |  |
| 2 | Vitvi11g00431\_t001 |  | \| \| |  | \| \| |  |  |  |  |  |  |  |  |
| 2 | Vitvi11g00433\_t001 |  | Vitvi04g00459\_t001 |  | \| \| |  |  |  |  |  |  |  |  |
| 2 | Vitvi11g04097\_t001 |  | \| \| |  | \| \| |  |  |  |  |  |  |  |  |
| 2 | Vitvi11g04098\_t001 |  | \| \| |  | \| \| |  |  |  |  |  |  |  |  |
| 2 | Vitvi11g00435\_t001 |  | \| \| |  | \| \| |  |  |  |  |  |  |  |  |
| 2 | Vitvi11g01427\_t001 |  | \| \| |  | \| \| |  |  |  |  |  |  |  |  |
| 2 | Vitvi11g04099\_t001 |  | Vitvi04g01894\_t001 |  | Vitvi09g04170\_t001 |  |  |  |  |  |  |  |  |
| 2 | Vitvi11g00436\_t001 |  | \| \| |  | Vitvi09g00531\_t001 |  |  |  |  |  |  |  |  |
| 2 | Vitvi11g00437\_t001 |  | \| \| |  | \| \| |  |  |  |  |  |  |  |  |
| 2 | Vitvi11g00438\_t001 |  | \| \| |  | Vitvi09g00539\_t001 |  |  |  |  |  |  |  |  |
| 2 | Vitvi11g00439\_t001 |  | \| \| |  | \| \| |  |  |  |  |  |  |  |  |
| 2 | Vitvi11g00440\_t001 |  | \| \| |  | \| \| |  |  |  |  |  |  |  |  |
| 2 | Vitvi11g01429\_t001 |  | Vitvi04g01898\_t001 |  | Vitvi09g01654\_t001 |  |  |  |  |  |  |  |  |
| 2 | Vitvi11g00442\_t001 |  | \| \| |  | \| \| |  |  |  |  |  |  |  |  |
| 2 | Vitvi11g04100\_t001 |  | \| \| |  | \| \| |  |  |  |  |  |  |  |  |
| 2 | Vitvi11g00443\_t001 |  | \| \| |  | Vitvi09g00559\_t001 |  |  |  |  |  |  |  |  |
| 2 | Vitvi11g00444\_t001 |  | \| \| |  | \| \| |  |  |  |  |  |  |  |  |
| 2 | Vitvi11g04101\_t001 |  | \| \| |  | \| \| |  |  |  |  |  |  |  |  |
| 2 | Vitvi11g00445\_t001 |  | \| \| |  | \| \| |  |  |  |  |  |  |  |  |
| 2 | Vitvi11g00446\_t001 |  | \| \| |  | \| \| |  |  |  |  |  |  |  |  |
| 2 | Vitvi11g00447\_t001 |  | \| \| |  | \| \| |  |  |  |  |  |  |  |  |
| 2 | Vitvi11g00448\_t001 |  | \| \| |  | \| \| |  |  |  |  |  |  |  |  |
| 2 | Vitvi11g00449\_t001 |  | \| \| |  | \| \| |  |  |  |  |  |  |  |  |
| 2 | Vitvi11g00450\_t001 |  | Vitvi04g01899\_t001 |  | Vitvi09g04178\_t001 |  |  |  |  |  |  |  |  |
| 2 | Vitvi11g00451\_t001 |  | Vitvi04g00466\_t001 |  | \| \| |  |  |  |  |  |  |  |  |
| 2 | Vitvi11g04102\_t001 |  | Vitvi04g00467\_t001 |  | Vitvi09g00573\_t001 |  |  |  |  |  |  |  |  |
| 2 | Vitvi11g01432\_t001 |  | \| \| |  | \| \| |  |  |  |  |  |  |  |  |
| 2 | Vitvi11g01433\_t002 |  | \| \| |  | Vitvi09g00576\_t001 |  |  |  |  |  |  |  |  |
| 2 | Vitvi11g00452\_t001 |  | \| \| |  | \| \| |  |  |  |  |  |  |  |  |
| 2 | Vitvi11g00453\_t001 |  | \| \| |  | \| \| |  |  |  |  |  |  |  |  |
| 2 | Vitvi11g01434\_t001 |  | \| \| |  | \| \| |  |  |  |  |  |  |  |  |
| 2 | Vitvi11g04103\_t001 |  | \| \| |  | \| \| |  |  |  |  |  |  |  |  |
| 2 | Vitvi11g00454\_t002 |  | Vitvi04g04121\_t001 |  | \| \| |  |  |  |  |  |  |  |  |
| 2 | Vitvi11g00456\_t001 |  | \| \| |  | \| \| |  |  |  |  |  |  |  |  |
| 2 | Vitvi11g04104\_t001 |  | \| \| |  | \| \| |  |  |  |  |  |  |  |  |
| 2 | Vitvi11g01435\_t001 |  | Vitvi04g00471\_t001 |  | \| \| |  |  |  |  |  |  |  |  |
| 2 | Vitvi11g00458\_t001 |  | \| \| |  | \| \| |  |  |  |  |  |  |  |  |
| 2 | Vitvi11g00459\_t001 |  | \| \| |  | \| \| |  |  |  |  |  |  |  |  |
| 2 | Vitvi11g00460\_t001 |  | \| \| |  | \| \| |  |  |  |  |  |  |  |  |
| 2 | Vitvi11g00461\_t001 |  | \| \| |  | \| \| |  |  |  |  |  |  |  |  |
| 2 | Vitvi11g00463\_t001 |  | \| \| |  | \| \| |  |  |  |  |  |  |  |  |
| 2 | Vitvi11g00464\_t001 |  | \| \| |  | \| \| |  |  |  |  |  |  |  |  |
| 2 | Vitvi11g04105\_t003 |  | Vitvi04g01902\_t001 |  | \| \| |  |  |  |  |  |  |  |  |
| 2 | Vitvi11g04106\_t001 |  | \| \| |  | \| \| |  |  |  |  |  |  |  |  |
| 2 | Vitvi11g04107\_t001 |  | \| \| |  | \| \| |  |  |  |  |  |  |  |  |
| 2 | Vitvi11g01442\_t001 |  | \| \| |  | \| \| |  |  |  |  |  |  |  |  |
| 2 | Vitvi11g04108\_t001 |  | \| \| |  | \| \| |  |  |  |  |  |  |  |  |
| 2 | Vitvi11g00466\_t001 |  | Vitvi04g00476\_t001 |  | Vitvi09g00591\_t001 |  |  |  |  |  |  |  |  |
| 2 | Vitvi11g04109\_t001 |  | \| \| |  | \| \| |  |  |  |  |  |  |  |  |
| 2 | Vitvi11g00467\_t001 |  | Vitvi04g00479\_t001 |  | Vitvi09g00593\_t001 |  |  |  |  |  |  |  |  |
| 2 | Vitvi11g00468\_t001 |  | \| \| |  | \| \| |  |  |  |  |  |  |  |  |
| 2 | Vitvi11g00469\_t001 |  | Vitvi04g00480\_t001 |  | Vitvi09g00595\_t001 |  |  |  |  |  |  |  |  |
| 2 | Vitvi11g00471\_t001 |  | \| \| |  | \| \| |  |  |  |  |  |  |  |  |
| 2 | Vitvi11g00472\_t001 |  | \| \| |  | Vitvi09g04184\_t001 |  |  |  |  |  |  |  |  |
| 2 | Vitvi11g00473\_t001 |  | \| \| |  | \| \| |  |  |  |  |  |  |  |  |
| 2 | Vitvi11g00474\_t001 |  | Vitvi04g00481\_t003 |  | Vitvi09g04185\_t001 |  |  |  |  |  |  |  |  |
| 2 | Vitvi11g00475\_t001 |  | Vitvi04g00482\_t001 |  | \| \| |  |  |  |  |  |  |  |  |
| 2 | Vitvi11g04110\_t001 |  | \| \| |  | \| \| |  |  |  |  |  |  |  |  |
| 2 | Vitvi11g01443\_t001 |  | \| \| |  | Vitvi09g00600\_t001 |  |  |  |  |  |  |  |  |
| 2 | Vitvi11g00476\_t003 |  | \| \| |  | \| \| |  |  |  |  |  |  |  |  |
| 2 | Vitvi11g04111\_t001 |  | \| \| |  | \| \| |  |  |  |  |  |  |  |  |
| 2 | Vitvi11g00477\_t001 |  | \| \| |  | Vitvi09g00601\_t001 |  |  |  |  |  |  |  |  |
| 2 | Vitvi11g00478\_t001 |  | \| \| |  | \| \| |  |  |  |  |  |  |  |  |
| 2 | Vitvi11g00479\_t001 |  | \| \| |  | Vitvi09g00603\_t001 |  |  |  |  |  |  |  |  |
| 2 | Vitvi11g00480\_t001 |  | Vitvi04g00483\_t002 |  | \| \| |  |  |  |  |  |  |  |  |
| 2 | Vitvi11g00481\_t001 |  | \| \| |  | \| \| |  |  |  |  |  |  |  |  |
| 2 | Vitvi11g00482\_t001 |  | \| \| |  | \| \| |  |  |  |  |  |  |  |  |
| 2 | Vitvi11g01444\_t001 |  | \| \| |  | \| \| |  |  |  |  |  |  |  |  |
| 2 | Vitvi11g00483\_t002 |  | \| \| |  | \| \| |  |  |  |  |  |  |  |  |
| 2 | Vitvi11g00484\_t001 |  | Vitvi04g00487\_t001 |  | \| \| |  |  |  |  |  |  |  |  |
| 2 | Vitvi11g01445\_t001 |  | \| \| |  | Vitvi09g01664\_t001 |  |  |  |  |  |  |  |  |
| 2 | Vitvi11g00485\_t001 |  | \| \| |  | \| \| |  |  |  |  |  |  |  |  |
| 2 | Vitvi11g00486\_t001 |  | \| \| |  | \| \| |  |  |  |  |  |  |  |  |
| 2 | Vitvi11g01446\_t001 |  | \| \| |  | \| \| |  |  |  |  |  |  |  |  |
| 2 | Vitvi11g00487\_t001 |  | \| \| |  | \| \| |  |  |  |  |  |  |  |  |
| 2 | Vitvi11g00488\_t001 |  | \| \| |  | \| \| |  |  |  |  |  |  |  |  |
| 2 | Vitvi11g00489\_t001 |  | \| \| |  | \| \| |  |  |  |  |  |  |  |  |
| 2 | Vitvi11g00492\_t001 |  | \| \| |  | \| \| |  |  |  |  |  |  |  |  |
| 2 | Vitvi11g00493\_t001 |  | \| \| |  | \| \| |  |  |  |  |  |  |  |  |
| 2 | Vitvi11g00494\_t001 |  | \| \| |  | \| \| |  |  |  |  |  |  |  |  |
| 2 | Vitvi11g00495\_t002 |  | \| \| |  | Vitvi09g00620\_t001 |  |  |  |  |  |  |  |  |
| 2 | Vitvi11g00496\_t001 |  | \| \| |  | \| \| |  |  |  |  |  |  |  |  |
| 2 | Vitvi11g04112\_t001 |  | \| \| |  | \| \| |  |  |  |  |  |  |  |  |
| 2 | Vitvi11g00497\_t001 |  | Vitvi04g00493\_t001 |  | \| \| |  |  |  |  |  |  |  |  |
| 2 | Vitvi11g00498\_t001 |  | \| \| |  | \| \| |  |  |  |  |  |  |  |  |
| 2 | Vitvi11g00499\_t001 |  | \| \| |  | \| \| |  |  |  |  |  |  |  |  |
| 2 | Vitvi11g00500\_t001 |  | \| \| |  | \| \| |  |  |  |  |  |  |  |  |
| 2 | Vitvi11g00501\_t001 |  | \| \| |  | \| \| |  |  |  |  |  |  |  |  |
| 2 | Vitvi11g00502\_t001 |  | \| \| |  | \| \| |  |  |  |  |  |  |  |  |
| 2 | Vitvi11g00503\_t002 |  | \| \| |  | \| \| |  |  |  |  |  |  |  |  |
| 2 | Vitvi11g00505\_t001 |  | Vitvi04g00494\_t001 |  | \| \| |  |  |  |  |  |  |  |  |
| 2 | Vitvi11g01447\_t001 |  | Vitvi04g00495\_t001 |  | Vitvi09g00625\_t001 |  |  |  |  |  |  |  |  |
| 2 | Vitvi11g00506\_t001 |  | \| \| |  | \| \| |  |  |  |  |  |  |  |  |
| 2 | Vitvi11g01448\_t001 |  | \| \| |  | Vitvi09g00630\_t001 |  |  |  |  |  |  |  |  |
| 1 | Vitvi11g00508\_t001 |  | \| \| |  |  |  |  |  |  |  |  |  |  |
| 1 | Vitvi11g00512\_t001 |  | \| \| |  |  |  |  |  |  |  |  |  |  |
| 1 | Vitvi11g00513\_t001 |  | \| \| |  |  |  |  |  |  |  |  |  |  |
| 1 | Vitvi11g00514\_t001 |  | \| \| |  |  |  |  |  |  |  |  |  |  |
| 1 | Vitvi11g00515\_t001 |  | \| \| |  |  |  |  |  |  |  |  |  |  |
| 1 | Vitvi11g00517\_t001 |  | \| \| |  |  |  |  |  |  |  |  |  |  |
| 1 | Vitvi11g00518\_t001 |  | Vitvi04g00501\_t001 |  |  |  |  |  |  |  |  |  |  |
| 1 | Vitvi11g04113\_t001 |  | Vitvi04g00503\_t001 |  |  |  |  |  |  |  |  |  |  |
| 1 | Vitvi11g04114\_t001 |  | \| \| |  |  |  |  |  |  |  |  |  |  |
| 1 | Vitvi11g04115\_t001 |  | \| \| |  |  |  |  |  |  |  |  |  |  |
| 1 | Vitvi11g04116\_t001 |  | \| \| |  |  |  |  |  |  |  |  |  |  |
| 1 | Vitvi11g04117\_t001 |  | \| \| |  |  |  |  |  |  |  |  |  |  |
| 1 | Vitvi11g00521\_t001 |  | Vitvi04g00505\_t001 |  |  |  |  |  |  |  |  |  |  |
| 1 | Vitvi11g01454\_t001 |  | \| \| |  |  |  |  |  |  |  |  |  |  |
| 1 | Vitvi11g00523\_t001 |  | \| \| |  |  |  |  |  |  |  |  |  |  |
| 1 | Vitvi11g00524\_t001 |  | \| \| |  |  |  |  |  |  |  |  |  |  |
| 1 | Vitvi11g00525\_t001 |  | \| \| |  |  |  |  |  |  |  |  |  |  |
| 1 | Vitvi11g00526\_t001 |  | \| \| |  |  |  |  |  |  |  |  |  |  |
| 1 | Vitvi11g00528\_t001 |  | Vitvi04g00507\_t001 |  |  |  |  |  |  |  |  |  |  |
| 1 | Vitvi11g04118\_t001 |  | \| \| |  |  |  |  |  |  |  |  |  |  |
| 1 | Vitvi11g04119\_t001 |  | \| \| |  |  |  |  |  |  |  |  |  |  |
| 1 | Vitvi11g04120\_t001 |  | \| \| |  |  |  |  |  |  |  |  |  |  |
| 1 | Vitvi11g04121\_t001 |  | \| \| |  |  |  |  |  |  |  |  |  |  |
| 1 | Vitvi11g01457\_t001 |  | \| \| |  |  |  |  |  |  |  |  |  |  |
| 1 | Vitvi11g00531\_t001 |  | \| \| |  |  |  |  |  |  |  |  |  |  |
| 1 | Vitvi11g04122\_t001 |  | \| \| |  |  |  |  |  |  |  |  |  |  |
| 1 | Vitvi11g01458\_t001 |  | \| \| |  |  |  |  |  |  |  |  |  |  |
| 1 | Vitvi11g00532\_t001 |  | \| \| |  |  |  |  |  |  |  |  |  |  |
| 1 | Vitvi11g04123\_t001 |  | \| \| |  |  |  |  |  |  |  |  |  |  |
| 1 | Vitvi11g04124\_t001 |  | \| \| |  |  |  |  |  |  |  |  |  |  |
| 1 | Vitvi11g04125\_t001 |  | \| \| |  |  |  |  |  |  |  |  |  |  |
| 1 | Vitvi11g04126\_t001 |  | \| \| |  |  |  |  |  |  |  |  |  |  |
| 1 | Vitvi11g04127\_t001 |  | \| \| |  |  |  |  |  |  |  |  |  |  |
| 1 | Vitvi11g04128\_t001 |  | \| \| |  |  |  |  |  |  |  |  |  |  |
| 1 | Vitvi11g00535\_t001 |  | \| \| |  |  |  |  |  |  |  |  |  |  |
| 1 | Vitvi11g00538\_t001 |  | Vitvi04g01910\_t001 |  |  |  |  |  |  |  |  |  |  |
| 1 | Vitvi11g00539\_t001 |  | \| \| |  |  |  |  |  |  |  |  |  |  |
| 1 | Vitvi11g04129\_t001 |  | \| \| |  |  |  |  |  |  |  |  |  |  |
| 1 | Vitvi11g00542\_t001 |  | Vitvi04g00508\_t001 |  |  |  |  |  |  |  |  |  |  |
| 1 | Vitvi11g04130\_t001 |  | \| \| |  |  |  |  |  |  |  |  |  |  |
| 1 | Vitvi11g04131\_t001 |  | \| \| |  |  |  |  |  |  |  |  |  |  |
| 1 | Vitvi11g00543\_t001 |  | \| \| |  |  |  |  |  |  |  |  |  |  |
| 1 | Vitvi11g00545\_t001 |  | \| \| |  |  |  |  |  |  |  |  |  |  |
| 1 | Vitvi11g00547\_t001 |  | Vitvi04g00509\_t001 |  |  |  |  |  |  |  |  |  |  |
| 2 | Vitvi11g00548\_t001 |  | Vitvi04g00532\_t001 |  | Vitvi04g00585\_t001 |  |  |  |  |  |  |  |  |
| 1 | Vitvi11g00549\_t001 |  |  |  | Vitvi04g00584\_t001 |  |  |  |  |  |  |  |  |
| 1 | Vitvi11g00550\_t001 |  |  |  | \| \| |  |  |  |  |  |  |  |  |
| 1 | Vitvi11g00552\_t002 |  |  |  | \| \| |  |  |  |  |  |  |  |  |
| 1 | Vitvi11g00554\_t001 |  |  |  | \| \| |  |  |  |  |  |  |  |  |
| 1 | Vitvi11g00555\_t001 |  |  |  | \| \| |  |  |  |  |  |  |  |  |
| 1 | Vitvi11g04132\_t001 |  |  |  | \| \| |  |  |  |  |  |  |  |  |
| 1 | Vitvi11g00557\_t001 |  |  |  | \| \| |  |  |  |  |  |  |  |  |
| 2 | Vitvi11g00560\_t001 |  | Vitvi09g00693\_t001 |  | \| \| |  |  |  |  |  |  |  |  |
| 2 | Vitvi11g00561\_t002 |  | Vitvi09g00720\_t001 |  | \| \| |  |  |  |  |  |  |  |  |
| 2 | Vitvi11g00564\_t001 |  | \| \| |  | \| \| |  |  |  |  |  |  |  |  |
| 2 | Vitvi11g00565\_t001 |  | \| \| |  | \| \| |  |  |  |  |  |  |  |  |
| 2 | Vitvi11g04133\_t001 |  | \| \| |  | \| \| |  |  |  |  |  |  |  |  |
| 2 | Vitvi11g00566\_t001 |  | \| \| |  | \| \| |  |  |  |  |  |  |  |  |
| 2 | Vitvi11g04134\_t001 |  | \| \| |  | \| \| |  |  |  |  |  |  |  |  |
| 2 | Vitvi11g00567\_t001 |  | \| \| |  | \| \| |  |  |  |  |  |  |  |  |
| 2 | Vitvi11g01463\_t001 |  | \| \| |  | Vitvi04g01925\_t001 |  |  |  |  |  |  |  |  |
| 2 | Vitvi11g04135\_t001 |  | \| \| |  | \| \| |  |  |  |  |  |  |  |  |
| 2 | Vitvi11g00568\_t001 |  | \| \| |  | \| \| |  |  |  |  |  |  |  |  |
| 2 | Vitvi11g01464\_t001 |  | \| \| |  | \| \| |  |  |  |  |  |  |  |  |
| 2 | Vitvi11g00570\_t001 |  | \| \| |  | \| \| |  |  |  |  |  |  |  |  |
| 2 | Vitvi11g00571\_t002 |  | \| \| |  | \| \| |  |  |  |  |  |  |  |  |
| 2 | Vitvi11g04136\_t001 |  | \| \| |  | \| \| |  |  |  |  |  |  |  |  |
| 2 | Vitvi11g01465\_t002 |  | \| \| |  | \| \| |  |  |  |  |  |  |  |  |
| 2 | Vitvi11g00573\_t001 |  | \| \| |  | \| \| |  |  |  |  |  |  |  |  |
| 2 | Vitvi11g00574\_t001 |  | Vitvi09g00734\_t001 |  | \| \| |  |  |  |  |  |  |  |  |
| 2 | Vitvi11g00575\_t001 |  | \| \| |  | Vitvi04g00565\_t001 |  |  |  |  |  |  |  |  |
| 2 | Vitvi11g00576\_t001 |  | \| \| |  | \| \| |  |  |  |  |  |  |  |  |
| 2 | Vitvi11g00577\_t001 |  | \| \| |  | \| \| |  |  |  |  |  |  |  |  |
| 2 | Vitvi11g00578\_t001 |  | \| \| |  | \| \| |  |  |  |  |  |  |  |  |
| 2 | Vitvi11g00579\_t001 |  | \| \| |  | Vitvi04g00559\_t001 |  |  |  |  |  |  |  |  |
| 2 | Vitvi11g00580\_t001 |  | Vitvi09g00759\_t001 |  | \| \| |  |  |  |  |  |  |  |  |
| 2 | Vitvi11g00581\_t001 |  | Vitvi09g00760\_t001 |  | \| \| |  |  |  |  |  |  |  |  |
| 2 | Vitvi11g00582\_t001 |  | Vitvi09g00772\_t001 |  | Vitvi04g00557\_t001 |  |  |  |  |  |  |  |  |
| 2 | Vitvi11g00583\_t001 |  | \| \| |  | \| \| |  |  |  |  |  |  |  |  |
| 2 | Vitvi11g04137\_t001 |  | \| \| |  | \| \| |  |  |  |  |  |  |  |  |
| 2 | Vitvi11g00584\_t001 |  | Vitvi09g00773\_t001 |  | Vitvi04g00556\_t001 |  |  |  |  |  |  |  |  |
| 1 | Vitvi11g00585\_t001 |  |  |  | \| \| |  |  |  |  |  |  |  |  |
| 1 | Vitvi11g01466\_t001 |  |  |  | \| \| |  |  |  |  |  |  |  |  |
| 1 | Vitvi11g01467\_t001 |  |  |  | \| \| |  |  |  |  |  |  |  |  |
| 1 | Vitvi11g00586\_t001 |  |  |  | \| \| |  |  |  |  |  |  |  |  |
| 1 | Vitvi11g00587\_t001 |  |  |  | \| \| |  |  |  |  |  |  |  |  |
| 1 | Vitvi11g01468\_t001 |  |  |  | \| \| |  |  |  |  |  |  |  |  |
| 1 | Vitvi11g04138\_t001 |  |  |  | \| \| |  |  |  |  |  |  |  |  |
| 1 | Vitvi11g00588\_t001 |  |  |  | Vitvi04g00555\_t001 |  |  |  |  |  |  |  |  |
| 1 | Vitvi11g04139\_t003 |  |  |  | \| \| |  |  |  |  |  |  |  |  |
| 1 | Vitvi11g00590\_t001 |  |  |  | Vitvi04g00554\_t001 |  |  |  |  |  |  |  |  |
| 1 | Vitvi11g00591\_t001 |  |  |  | \| \| |  |  |  |  |  |  |  |  |
| 1 | Vitvi11g04140\_t001 |  |  |  | \| \| |  |  |  |  |  |  |  |  |
| 1 | Vitvi11g00593\_t001 |  |  |  | \| \| |  |  |  |  |  |  |  |  |
| 1 | Vitvi11g00595\_t001 |  |  |  | \| \| |  |  |  |  |  |  |  |  |
| 1 | Vitvi11g01471\_t001 |  |  |  | \| \| |  |  |  |  |  |  |  |  |
| 1 | Vitvi11g04141\_t001 |  |  |  | \| \| |  |  |  |  |  |  |  |  |
| 1 | Vitvi11g01473\_t001 |  |  |  | \| \| |  |  |  |  |  |  |  |  |
| 1 | Vitvi11g04142\_t001 |  |  |  | \| \| |  |  |  |  |  |  |  |  |
| 1 | Vitvi11g00597\_t001 |  |  |  | \| \| |  |  |  |  |  |  |  |  |
| 1 | Vitvi11g00598\_t001 |  |  |  | \| \| |  |  |  |  |  |  |  |  |
| 1 | Vitvi11g00599\_t002 |  |  |  | \| \| |  |  |  |  |  |  |  |  |
| 1 | Vitvi11g01474\_t001 |  |  |  | Vitvi04g01918\_t001 |  |  |  |  |  |  |  |  |
| 1 | Vitvi11g04143\_t001 |  |  |  | \| \| |  |  |  |  |  |  |  |  |
| 1 | Vitvi11g00600\_t001 |  |  |  | \| \| |  |  |  |  |  |  |  |  |
| 1 | Vitvi11g01475\_t001 |  |  |  | \| \| |  |  |  |  |  |  |  |  |
| 1 | Vitvi11g04144\_t001 |  |  |  | \| \| |  |  |  |  |  |  |  |  |
| 1 | Vitvi11g00601\_t002 |  |  |  | Vitvi04g00546\_t001 |  |  |  |  |  |  |  |  |
| 0 | Vitvi11g04145\_t001 |  |  |  |  |  |  |  |  |  |  |  |  |
| 0 | Vitvi11g00603\_t001 |  |  |  |  |  |  |  |  |  |  |  |  |
| 0 | Vitvi11g00604\_t001 |  |  |  |  |  |  |  |  |  |  |  |  |
| 0 | Vitvi11g04146\_t001 |  |  |  |  |  |  |  |  |  |  |  |  |
| 0 | Vitvi11g04147\_t001 |  |  |  |  |  |  |  |  |  |  |  |  |
| 0 | Vitvi11g00606\_t001 |  |  |  |  |  |  |  |  |  |  |  |  |
| 0 | Vitvi11g04148\_t001 |  |  |  |  |  |  |  |  |  |  |  |  |
| 0 | Vitvi11g04149\_t001 |  |  |  |  |  |  |  |  |  |  |  |  |
| 0 | Vitvi11g04150\_t001 |  |  |  |  |  |  |  |  |  |  |  |  |
| 0 | Vitvi11g00608\_t001 |  |  |  |  |  |  |  |  |  |  |  |  |
| 0 | Vitvi11g04151\_t001 |  |  |  |  |  |  |  |  |  |  |  |  |
| 0 | Vitvi11g00612\_t001 |  |  |  |  |  |  |  |  |  |  |  |  |
| 0 | Vitvi11g04152\_t001 |  |  |  |  |  |  |  |  |  |  |  |  |
| 0 | Vitvi11g00613\_t001 |  |  |  |  |  |  |  |  |  |  |  |  |
| 0 | Vitvi11g00614\_t003 |  |  |  |  |  |  |  |  |  |  |  |  |
| 0 | Vitvi11g04153\_t001 |  |  |  |  |  |  |  |  |  |  |  |  |
| 0 | Vitvi11g00615\_t001 |  |  |  |  |  |  |  |  |  |  |  |  |
| 0 | Vitvi11g01479\_t001 |  |  |  |  |  |  |  |  |  |  |  |  |
| 0 | Vitvi11g00618\_t001 |  |  |  |  |  |  |  |  |  |  |  |  |
| 0 | Vitvi11g00619\_t001 |  |  |  |  |  |  |  |  |  |  |  |  |
| 0 | Vitvi11g00620\_t001 |  |  |  |  |  |  |  |  |  |  |  |  |
| 0 | Vitvi11g04154\_t001 |  |  |  |  |  |  |  |  |  |  |  |  |
| 0 | Vitvi11g04155\_t001 |  |  |  |  |  |  |  |  |  |  |  |  |
| 0 | Vitvi11g04156\_t001 |  |  |  |  |  |  |  |  |  |  |  |  |
| 0 | Vitvi11g00622\_t001 |  |  |  |  |  |  |  |  |  |  |  |  |
| 0 | Vitvi11g00623\_t001 |  |  |  |  |  |  |  |  |  |  |  |  |
| 0 | Vitvi11g00625\_t001 |  |  |  |  |  |  |  |  |  |  |  |  |
| 0 | Vitvi11g00626\_t001 |  |  |  |  |  |  |  |  |  |  |  |  |
| 0 | Vitvi11g01483\_t001 |  |  |  |  |  |  |  |  |  |  |  |  |
| 0 | Vitvi11g00631\_t001 |  |  |  |  |  |  |  |  |  |  |  |  |
| 0 | Vitvi11g00633\_t001 |  |  |  |  |  |  |  |  |  |  |  |  |
| 0 | Vitvi11g00634\_t001 |  |  |  |  |  |  |  |  |  |  |  |  |
| 0 | Vitvi11g04157\_t001 |  |  |  |  |  |  |  |  |  |  |  |  |
| 0 | Vitvi11g01486\_t001 |  |  |  |  |  |  |  |  |  |  |  |  |
| 0 | Vitvi11g01487\_t001 |  |  |  |  |  |  |  |  |  |  |  |  |
| 0 | Vitvi11g00635\_t001 |  |  |  |  |  |  |  |  |  |  |  |  |
| 0 | Vitvi11g00636\_t001 |  |  |  |  |  |  |  |  |  |  |  |  |
| 0 | Vitvi11g04158\_t001 |  |  |  |  |  |  |  |  |  |  |  |  |
| 0 | Vitvi11g00640\_t001 |  |  |  |  |  |  |  |  |  |  |  |  |
| 0 | Vitvi11g00641\_t001 |  |  |  |  |  |  |  |  |  |  |  |  |
| 0 | Vitvi11g04159\_t001 |  |  |  |  |  |  |  |  |  |  |  |  |
| 0 | Vitvi11g01488\_t001 |  |  |  |  |  |  |  |  |  |  |  |  |
| 0 | Vitvi11g04160\_t001 |  |  |  |  |  |  |  |  |  |  |  |  |
| 0 | Vitvi11g04161\_t001 |  |  |  |  |  |  |  |  |  |  |  |  |
| 0 | Vitvi11g04162\_t001 |  |  |  |  |  |  |  |  |  |  |  |  |
| 0 | Vitvi11g00651\_t001 |  |  |  |  |  |  |  |  |  |  |  |  |
| 0 | Vitvi11g00652\_t002 |  |  |  |  |  |  |  |  |  |  |  |  |
| 0 | Vitvi11g04163\_t001 |  |  |  |  |  |  |  |  |  |  |  |  |
| 0 | Vitvi11g00655\_t002 |  |  |  |  |  |  |  |  |  |  |  |  |
| 0 | Vitvi11g04164\_t001 |  |  |  |  |  |  |  |  |  |  |  |  |
| 0 | Vitvi11g00658\_t001 |  |  |  |  |  |  |  |  |  |  |  |  |
| 0 | Vitvi11g00659\_t001 |  |  |  |  |  |  |  |  |  |  |  |  |
| 0 | Vitvi11g00660\_t001 |  |  |  |  |  |  |  |  |  |  |  |  |
| 0 | Vitvi11g00661\_t003 |  |  |  |  |  |  |  |  |  |  |  |  |
| 0 | Vitvi11g04165\_t001 |  |  |  |  |  |  |  |  |  |  |  |  |
| 0 | Vitvi11g00666\_t001 |  |  |  |  |  |  |  |  |  |  |  |  |
| 0 | Vitvi11g00667\_t001 |  |  |  |  |  |  |  |  |  |  |  |  |
| 0 | Vitvi11g00668\_t001 |  |  |  |  |  |  |  |  |  |  |  |  |
| 0 | Vitvi11g04166\_t001 |  |  |  |  |  |  |  |  |  |  |  |  |
| 0 | Vitvi11g04167\_t001 |  |  |  |  |  |  |  |  |  |  |  |  |
| 0 | Vitvi11g01715\_t001 |  |  |  |  |  |  |  |  |  |  |  |  |
| 0 | Vitvi11g00671\_t001 |  |  |  |  |  |  |  |  |  |  |  |  |
| 0 | Vitvi11g00672\_t001 |  |  |  |  |  |  |  |  |  |  |  |  |
| 0 | Vitvi11g04168\_t001 |  |  |  |  |  |  |  |  |  |  |  |  |
| 1 | Vitvi11g00673\_t001 |  | Vitvi04g00586\_t001 |  |  |  |  |  |  |  |  |  |  |
| 1 | Vitvi11g04169\_t001 |  | \| \| |  |  |  |  |  |  |  |  |  |  |
| 1 | Vitvi11g04170\_t001 |  | \| \| |  |  |  |  |  |  |  |  |  |  |
| 1 | Vitvi11g04171\_t001 |  | \| \| |  |  |  |  |  |  |  |  |  |  |
| 1 | Vitvi11g00677\_t001 |  | \| \| |  |  |  |  |  |  |  |  |  |  |
| 1 | Vitvi11g00676\_t001 |  | \| \| |  |  |  |  |  |  |  |  |  |  |
| 1 | Vitvi11g04172\_t001 |  | \| \| |  |  |  |  |  |  |  |  |  |  |
| 1 | Vitvi11g00678\_t001 |  | \| \| |  |  |  |  |  |  |  |  |  |  |
| 1 | Vitvi11g04173\_t001 |  | \| \| |  |  |  |  |  |  |  |  |  |  |
| 1 | Vitvi11g00679\_t002 |  | \| \| |  |  |  |  |  |  |  |  |  |  |
| 1 | Vitvi11g00680\_t001 |  | \| \| |  |  |  |  |  |  |  |  |  |  |
| 1 | Vitvi11g00682\_t002 |  | \| \| |  |  |  |  |  |  |  |  |  |  |
| 1 | Vitvi11g00683\_t001 |  | \| \| |  |  |  |  |  |  |  |  |  |  |
| 1 | Vitvi11g00685\_t001 |  | \| \| |  |  |  |  |  |  |  |  |  |  |
| 1 | Vitvi11g00686\_t001 |  | \| \| |  |  |  |  |  |  |  |  |  |  |
| 1 | Vitvi11g00687\_t001 |  | \| \| |  |  |  |  |  |  |  |  |  |  |
| 1 | Vitvi11g00688\_t001 |  | \| \| |  |  |  |  |  |  |  |  |  |  |
| 1 | Vitvi11g00689\_t001 |  | \| \| |  |  |  |  |  |  |  |  |  |  |
| 1 | Vitvi11g00690\_t001 |  | \| \| |  |  |  |  |  |  |  |  |  |  |
| 1 | Vitvi11g00692\_t001 |  | \| \| |  |  |  |  |  |  |  |  |  |  |
| 1 | Vitvi11g00694\_t002 |  | Vitvi04g00596\_t001 |  |  |  |  |  |  |  |  |  |  |
| 1 | Vitvi11g00695\_t001 |  | \| \| |  |  |  |  |  |  |  |  |  |  |
| 1 | Vitvi11g04174\_t001 |  | \| \| |  |  |  |  |  |  |  |  |  |  |
| 1 | Vitvi11g00696\_t001 |  | \| \| |  |  |  |  |  |  |  |  |  |  |
| 1 | Vitvi11g00698\_t001 |  | \| \| |  |  |  |  |  |  |  |  |  |  |
| 1 | Vitvi11g00699\_t001 |  | \| \| |  |  |  |  |  |  |  |  |  |  |
| 1 | Vitvi11g00700\_t001 |  | Vitvi04g00600\_t001 |  |  |  |  |  |  |  |  |  |  |
| 1 | Vitvi11g00701\_t002 |  | \| \| |  |  |  |  |  |  |  |  |  |  |
| 1 | Vitvi11g00704\_t001 |  | \| \| |  |  |  |  |  |  |  |  |  |  |
| 1 | Vitvi11g04175\_t001 |  | \| \| |  |  |  |  |  |  |  |  |  |  |
| 1 | Vitvi11g04176\_t001 |  | Vitvi04g00601\_t001 |  |  |  |  |  |  |  |  |  |  |
| 1 | Vitvi11g04177\_t001 |  | \| \| |  |  |  |  |  |  |  |  |  |  |
| 1 | Vitvi11g00707\_t001 |  | \| \| |  |  |  |  |  |  |  |  |  |  |
| 1 | Vitvi11g00708\_t001 |  | Vitvi04g00602\_t001 |  |  |  |  |  |  |  |  |  |  |
| 1 | Vitvi11g00710\_t001 |  | \| \| |  |  |  |  |  |  |  |  |  |  |
| 1 | Vitvi11g00711\_t001 |  | \| \| |  |  |  |  |  |  |  |  |  |  |
| 1 | Vitvi11g00712\_t001 |  | \| \| |  |  |  |  |  |  |  |  |  |  |
| 1 | Vitvi11g00713\_t001 |  | \| \| |  |  |  |  |  |  |  |  |  |  |
| 1 | Vitvi11g01504\_t001 |  | \| \| |  |  |  |  |  |  |  |  |  |  |
| 1 | Vitvi11g04178\_t001 |  | \| \| |  |  |  |  |  |  |  |  |  |  |
| 1 | Vitvi11g01505\_t001 |  | Vitvi04g01929\_t001 |  |  |  |  |  |  |  |  |  |  |
| 1 | Vitvi11g00715\_t001 |  | Vitvi04g01930\_t001 |  |  |  |  |  |  |  |  |  |  |
| 1 | Vitvi11g00717\_t001 |  | \| \| |  |  |  |  |  |  |  |  |  |  |
| 1 | Vitvi11g00718\_t001 |  | \| \| |  |  |  |  |  |  |  |  |  |  |
| 1 | Vitvi11g00719\_t001 |  | \| \| |  |  |  |  |  |  |  |  |  |  |
| 1 | Vitvi11g00720\_t001 |  | \| \| |  |  |  |  |  |  |  |  |  |  |
| 1 | Vitvi11g01506\_t001 |  | \| \| |  |  |  |  |  |  |  |  |  |  |
| 1 | Vitvi11g01507\_t001 |  | \| \| |  |  |  |  |  |  |  |  |  |  |
| 1 | Vitvi11g00722\_t001 |  | \| \| |  |  |  |  |  |  |  |  |  |  |
| 1 | Vitvi11g00723\_t001 |  | Vitvi04g00609\_t001 |  |  |  |  |  |  |  |  |  |  |
| 0 | Vitvi11g04179\_t001 |  |  |  |  |  |  |  |  |  |  |  |  |
| 0 | Vitvi11g04180\_t001 |  |  |  |  |  |  |  |  |  |  |  |  |
| 0 | Vitvi11g00724\_t001 |  |  |  |  |  |  |  |  |  |  |  |  |
| 0 | Vitvi11g00725\_t001 |  |  |  |  |  |  |  |  |  |  |  |  |
| 0 | Vitvi11g00726\_t001 |  |  |  |  |  |  |  |  |  |  |  |  |
| 0 | Vitvi11g00727\_t001 |  |  |  |  |  |  |  |  |  |  |  |  |
| 0 | Vitvi11g00728\_t002 |  |  |  |  |  |  |  |  |  |  |  |  |
| 0 | Vitvi11g00730\_t001 |  |  |  |  |  |  |  |  |  |  |  |  |
| 0 | Vitvi11g04181\_t001 |  |  |  |  |  |  |  |  |  |  |  |  |
| 0 | Vitvi11g00735\_t001 |  |  |  |  |  |  |  |  |  |  |  |  |
| 0 | Vitvi11g04182\_t001 |  |  |  |  |  |  |  |  |  |  |  |  |
| 0 | Vitvi11g04183\_t001 |  |  |  |  |  |  |  |  |  |  |  |  |
| 0 | Vitvi11g00738\_t001 |  |  |  |  |  |  |  |  |  |  |  |  |
| 0 | Vitvi11g04184\_t001 |  |  |  |  |  |  |  |  |  |  |  |  |
| 0 | Vitvi11g00742\_t001 |  |  |  |  |  |  |  |  |  |  |  |  |
| 0 | Vitvi11g00744\_t001 |  |  |  |  |  |  |  |  |  |  |  |  |
| 0 | Vitvi11g00745\_t001 |  |  |  |  |  |  |  |  |  |  |  |  |
| 0 | Vitvi11g00746\_t001 |  |  |  |  |  |  |  |  |  |  |  |  |
| 0 | Vitvi11g04185\_t001 |  |  |  |  |  |  |  |  |  |  |  |  |
| 0 | Vitvi11g00748\_t001 |  |  |  |  |  |  |  |  |  |  |  |  |
| 0 | Vitvi11g04186\_t001 |  |  |  |  |  |  |  |  |  |  |  |  |
| 0 | Vitvi11g04187\_t001 |  |  |  |  |  |  |  |  |  |  |  |  |
| 0 | Vitvi11g00749\_t001 |  |  |  |  |  |  |  |  |  |  |  |  |
| 0 | Vitvi11g00751\_t001 |  |  |  |  |  |  |  |  |  |  |  |  |
| 0 | Vitvi11g00755\_t001 |  |  |  |  |  |  |  |  |  |  |  |  |
| 0 | Vitvi11g04188\_t001 |  |  |  |  |  |  |  |  |  |  |  |  |
| 0 | Vitvi11g00757\_t001 |  |  |  |  |  |  |  |  |  |  |  |  |
| 0 | Vitvi11g04189\_t001 |  |  |  |  |  |  |  |  |  |  |  |  |
| 0 | Vitvi11g04190\_t001 |  |  |  |  |  |  |  |  |  |  |  |  |
| 0 | Vitvi11g04191\_t001 |  |  |  |  |  |  |  |  |  |  |  |  |
| 0 | Vitvi11g04192\_t001 |  |  |  |  |  |  |  |  |  |  |  |  |
| 0 | Vitvi11g04193\_t001 |  |  |  |  |  |  |  |  |  |  |  |  |
| 0 | Vitvi11g04194\_t001 |  |  |  |  |  |  |  |  |  |  |  |  |
| 0 | Vitvi11g04195\_t001 |  |  |  |  |  |  |  |  |  |  |  |  |
| 0 | Vitvi11g04196\_t001 |  |  |  |  |  |  |  |  |  |  |  |  |
| 0 | Vitvi11g04197\_t001 |  |  |  |  |  |  |  |  |  |  |  |  |
| 0 | Vitvi11g04198\_t001 |  |  |  |  |  |  |  |  |  |  |  |  |
| 0 | Vitvi11g04199\_t001 |  |  |  |  |  |  |  |  |  |  |  |  |
| 0 | Vitvi11g00764\_t001 |  |  |  |  |  |  |  |  |  |  |  |  |
| 0 | Vitvi11g04200\_t001 |  |  |  |  |  |  |  |  |  |  |  |  |
| 0 | Vitvi11g04201\_t001 |  |  |  |  |  |  |  |  |  |  |  |  |
| 0 | Vitvi11g04202\_t001 |  |  |  |  |  |  |  |  |  |  |  |  |
| 0 | Vitvi11g01513\_t001 |  |  |  |  |  |  |  |  |  |  |  |  |
| 0 | Vitvi11g00766\_t001 |  |  |  |  |  |  |  |  |  |  |  |  |
| 0 | Vitvi11g00767\_t001 |  |  |  |  |  |  |  |  |  |  |  |  |
| 0 | Vitvi11g00770\_t001 |  |  |  |  |  |  |  |  |  |  |  |  |
| 0 | Vitvi11g00771\_t001 |  |  |  |  |  |  |  |  |  |  |  |  |
| 0 | Vitvi11g00776\_t001 |  |  |  |  |  |  |  |  |  |  |  |  |
| 0 | Vitvi11g00777\_t001 |  |  |  |  |  |  |  |  |  |  |  |  |
| 0 | Vitvi11g00778\_t001 |  |  |  |  |  |  |  |  |  |  |  |  |
| 0 | Vitvi11g01514\_t001 |  |  |  |  |  |  |  |  |  |  |  |  |
| 0 | Vitvi11g04203\_t001 |  |  |  |  |  |  |  |  |  |  |  |  |
| 0 | Vitvi11g04204\_t001 |  |  |  |  |  |  |  |  |  |  |  |  |
| 0 | Vitvi11g00783\_t001 |  |  |  |  |  |  |  |  |  |  |  |  |
| 0 | Vitvi11g01516\_t001 |  |  |  |  |  |  |  |  |  |  |  |  |
| 0 | Vitvi11g04205\_t001 |  |  |  |  |  |  |  |  |  |  |  |  |
| 0 | Vitvi11g01517\_t001 |  |  |  |  |  |  |  |  |  |  |  |  |
| 0 | Vitvi11g00786\_t001 |  |  |  |  |  |  |  |  |  |  |  |  |
| 0 | Vitvi11g00787\_t001 |  |  |  |  |  |  |  |  |  |  |  |  |
| 0 | Vitvi11g04206\_t001 |  |  |  |  |  |  |  |  |  |  |  |  |
| 0 | Vitvi11g00788\_t001 |  |  |  |  |  |  |  |  |  |  |  |  |
| 0 | Vitvi11g00795\_t001 |  |  |  |  |  |  |  |  |  |  |  |  |
| 0 | Vitvi11g00796\_t001 |  |  |  |  |  |  |  |  |  |  |  |  |
| 0 | Vitvi11g00798\_t001 |  |  |  |  |  |  |  |  |  |  |  |  |
| 0 | Vitvi11g00800\_t001 |  |  |  |  |  |  |  |  |  |  |  |  |
| 0 | Vitvi11g04207\_t001 |  |  |  |  |  |  |  |  |  |  |  |  |
| 0 | Vitvi11g04208\_t001 |  |  |  |  |  |  |  |  |  |  |  |  |
| 0 | Vitvi11g00805\_t001 |  |  |  |  |  |  |  |  |  |  |  |  |
| 0 | Vitvi11g00809\_t002 |  |  |  |  |  |  |  |  |  |  |  |  |
| 0 | Vitvi11g04209\_t001 |  |  |  |  |  |  |  |  |  |  |  |  |
| 0 | Vitvi11g00811\_t001 |  |  |  |  |  |  |  |  |  |  |  |  |
| 0 | Vitvi11g00812\_t001 |  |  |  |  |  |  |  |  |  |  |  |  |
| 0 | Vitvi11g00813\_t001 |  |  |  |  |  |  |  |  |  |  |  |  |
| 0 | Vitvi11g01522\_t001 |  |  |  |  |  |  |  |  |  |  |  |  |
| 0 | Vitvi11g00814\_t001 |  |  |  |  |  |  |  |  |  |  |  |  |
| 0 | Vitvi11g00815\_t001 |  |  |  |  |  |  |  |  |  |  |  |  |
| 0 | Vitvi11g00818\_t001 |  |  |  |  |  |  |  |  |  |  |  |  |
| 0 | Vitvi11g00819\_t001 |  |  |  |  |  |  |  |  |  |  |  |  |
| 0 | Vitvi11g00821\_t001 |  |  |  |  |  |  |  |  |  |  |  |  |
| 0 | Vitvi11g00822\_t001 |  |  |  |  |  |  |  |  |  |  |  |  |
| 0 | Vitvi11g00823\_t001 |  |  |  |  |  |  |  |  |  |  |  |  |
| 0 | Vitvi11g01523\_t001 |  |  |  |  |  |  |  |  |  |  |  |  |
| 0 | Vitvi11g00824\_t001 |  |  |  |  |  |  |  |  |  |  |  |  |
| 0 | Vitvi11g01525\_t001 |  |  |  |  |  |  |  |  |  |  |  |  |
| 0 | Vitvi11g00825\_t001 |  |  |  |  |  |  |  |  |  |  |  |  |
| 0 | Vitvi11g00826\_t001 |  |  |  |  |  |  |  |  |  |  |  |  |
| 0 | Vitvi11g00828\_t001 |  |  |  |  |  |  |  |  |  |  |  |  |
| 0 | Vitvi11g01527\_t001 |  |  |  |  |  |  |  |  |  |  |  |  |
| 0 | Vitvi11g04210\_t001 |  |  |  |  |  |  |  |  |  |  |  |  |
| 0 | Vitvi11g01528\_t001 |  |  |  |  |  |  |  |  |  |  |  |  |
| 0 | Vitvi11g04211\_t001 |  |  |  |  |  |  |  |  |  |  |  |  |
| 0 | Vitvi11g04212\_t001 |  |  |  |  |  |  |  |  |  |  |  |  |
| 0 | Vitvi11g00835\_t001 |  |  |  |  |  |  |  |  |  |  |  |  |
| 0 | Vitvi11g04213\_t001 |  |  |  |  |  |  |  |  |  |  |  |  |
| 0 | Vitvi11g04214\_t001 |  |  |  |  |  |  |  |  |  |  |  |  |
| 0 | Vitvi11g00838\_t001 |  |  |  |  |  |  |  |  |  |  |  |  |
| 0 | Vitvi11g00840\_t001 |  |  |  |  |  |  |  |  |  |  |  |  |
| 0 | Vitvi11g00841\_t001 |  |  |  |  |  |  |  |  |  |  |  |  |
| 0 | Vitvi11g00842\_t001 |  |  |  |  |  |  |  |  |  |  |  |  |
| 0 | Vitvi11g00843\_t001 |  |  |  |  |  |  |  |  |  |  |  |  |
| 0 | Vitvi11g00847\_t001 |  |  |  |  |  |  |  |  |  |  |  |  |
| 0 | Vitvi11g00848\_t001 |  |  |  |  |  |  |  |  |  |  |  |  |
| 0 | Vitvi11g01533\_t001 |  |  |  |  |  |  |  |  |  |  |  |  |
| 0 | Vitvi11g00851\_t001 |  |  |  |  |  |  |  |  |  |  |  |  |
| 0 | Vitvi11g04215\_t001 |  |  |  |  |  |  |  |  |  |  |  |  |
| 0 | Vitvi11g01536\_t001 |  |  |  |  |  |  |  |  |  |  |  |  |
| 0 | Vitvi11g00854\_t001 |  |  |  |  |  |  |  |  |  |  |  |  |
| 0 | Vitvi11g01537\_t001 |  |  |  |  |  |  |  |  |  |  |  |  |
| 0 | Vitvi11g04216\_t001 |  |  |  |  |  |  |  |  |  |  |  |  |
| 0 | Vitvi11g01539\_t001 |  |  |  |  |  |  |  |  |  |  |  |  |
| 0 | Vitvi11g04217\_t001 |  |  |  |  |  |  |  |  |  |  |  |  |
| 0 | Vitvi11g00864\_t001 |  |  |  |  |  |  |  |  |  |  |  |  |
| 0 | Vitvi11g00865\_t001 |  |  |  |  |  |  |  |  |  |  |  |  |
| 0 | Vitvi11g00869\_t001 |  |  |  |  |  |  |  |  |  |  |  |  |
| 0 | Vitvi11g00871\_t001 |  |  |  |  |  |  |  |  |  |  |  |  |
| 0 | Vitvi11g04218\_t001 |  |  |  |  |  |  |  |  |  |  |  |  |
| 0 | Vitvi11g04219\_t001 |  |  |  |  |  |  |  |  |  |  |  |  |
| 0 | Vitvi11g04220\_t001 |  |  |  |  |  |  |  |  |  |  |  |  |
| 0 | Vitvi11g00875\_t001 |  |  |  |  |  |  |  |  |  |  |  |  |
| 0 | Vitvi11g00878\_t002 |  |  |  |  |  |  |  |  |  |  |  |  |
| 0 | Vitvi11g04221\_t001 |  |  |  |  |  |  |  |  |  |  |  |  |
| 0 | Vitvi11g04222\_t001 |  |  |  |  |  |  |  |  |  |  |  |  |
| 0 | Vitvi11g04223\_t001 |  |  |  |  |  |  |  |  |  |  |  |  |
| 0 | Vitvi11g04224\_t001 |  |  |  |  |  |  |  |  |  |  |  |  |
| 0 | Vitvi11g04225\_t001 |  |  |  |  |  |  |  |  |  |  |  |  |
| 0 | Vitvi11g04226\_t001 |  |  |  |  |  |  |  |  |  |  |  |  |
| 0 | Vitvi11g04227\_t001 |  |  |  |  |  |  |  |  |  |  |  |  |
| 0 | Vitvi11g04228\_t001 |  |  |  |  |  |  |  |  |  |  |  |  |
| 0 | Vitvi11g04229\_t001 |  |  |  |  |  |  |  |  |  |  |  |  |
| 0 | Vitvi11g04230\_t001 |  |  |  |  |  |  |  |  |  |  |  |  |
| 0 | Vitvi11g04231\_t001 |  |  |  |  |  |  |  |  |  |  |  |  |
| 0 | Vitvi11g04232\_t001 |  |  |  |  |  |  |  |  |  |  |  |  |
| 0 | Vitvi11g00890\_t001 |  |  |  |  |  |  |  |  |  |  |  |  |
| 0 | Vitvi11g00891\_t001 |  |  |  |  |  |  |  |  |  |  |  |  |
| 0 | Vitvi11g04233\_t001 |  |  |  |  |  |  |  |  |  |  |  |  |
| 0 | Vitvi11g04234\_t001 |  |  |  |  |  |  |  |  |  |  |  |  |
| 0 | Vitvi11g04235\_t001 |  |  |  |  |  |  |  |  |  |  |  |  |
| 0 | Vitvi11g04236\_t001 |  |  |  |  |  |  |  |  |  |  |  |  |
| 0 | Vitvi11g04237\_t001 |  |  |  |  |  |  |  |  |  |  |  |  |
| 0 | Vitvi11g00893\_t001 |  |  |  |  |  |  |  |  |  |  |  |  |
| 0 | Vitvi11g04238\_t001 |  |  |  |  |  |  |  |  |  |  |  |  |
| 0 | Vitvi11g04239\_t001 |  |  |  |  |  |  |  |  |  |  |  |  |
| 0 | Vitvi11g00896\_t001 |  |  |  |  |  |  |  |  |  |  |  |  |
| 0 | Vitvi11g04240\_t001 |  |  |  |  |  |  |  |  |  |  |  |  |
| 0 | Vitvi11g04241\_t001 |  |  |  |  |  |  |  |  |  |  |  |  |
| 0 | Vitvi11g00900\_t001 |  |  |  |  |  |  |  |  |  |  |  |  |
| 0 | Vitvi11g04242\_t001 |  |  |  |  |  |  |  |  |  |  |  |  |
| 0 | Vitvi11g04243\_t001 |  |  |  |  |  |  |  |  |  |  |  |  |
| 0 | Vitvi11g00901\_t001 |  |  |  |  |  |  |  |  |  |  |  |  |
| 0 | Vitvi11g00903\_t001 |  |  |  |  |  |  |  |  |  |  |  |  |
| 0 | Vitvi11g00904\_t001 |  |  |  |  |  |  |  |  |  |  |  |  |
| 0 | Vitvi11g00906\_t001 |  |  |  |  |  |  |  |  |  |  |  |  |
| 0 | Vitvi11g00907\_t001 |  |  |  |  |  |  |  |  |  |  |  |  |
| 0 | Vitvi11g00909\_t001 |  |  |  |  |  |  |  |  |  |  |  |  |
| 0 | Vitvi11g04244\_t001 |  |  |  |  |  |  |  |  |  |  |  |  |
| 0 | Vitvi11g00910\_t001 |  |  |  |  |  |  |  |  |  |  |  |  |
| 0 | Vitvi11g04245\_t001 |  |  |  |  |  |  |  |  |  |  |  |  |
| 0 | Vitvi11g00912\_t001 |  |  |  |  |  |  |  |  |  |  |  |  |
| 0 | Vitvi11g04246\_t001 |  |  |  |  |  |  |  |  |  |  |  |  |
| 0 | Vitvi11g00916\_t001 |  |  |  |  |  |  |  |  |  |  |  |  |
| 0 | Vitvi11g00919\_t001 |  |  |  |  |  |  |  |  |  |  |  |  |
| 0 | Vitvi11g04247\_t001 |  |  |  |  |  |  |  |  |  |  |  |  |
| 0 | Vitvi11g00920\_t001 |  |  |  |  |  |  |  |  |  |  |  |  |
| 0 | Vitvi11g00921\_t001 |  |  |  |  |  |  |  |  |  |  |  |  |
| 0 | Vitvi11g00923\_t001 |  |  |  |  |  |  |  |  |  |  |  |  |
| 0 | Vitvi11g00924\_t001 |  |  |  |  |  |  |  |  |  |  |  |  |
| 0 | Vitvi11g00926\_t001 |  |  |  |  |  |  |  |  |  |  |  |  |
| 0 | Vitvi11g00927\_t002 |  |  |  |  |  |  |  |  |  |  |  |  |
| 0 | Vitvi11g00928\_t001.1.6037826a |  |  |  |  |  |  |  |  |  |  |  |  |
| 0 | Vitvi11g00929\_t001 |  |  |  |  |  |  |  |  |  |  |  |  |
| 0 | Vitvi11g00930\_t001 |  |  |  |  |  |  |  |  |  |  |  |  |
| 0 | Vitvi11g00931\_t001 |  |  |  |  |  |  |  |  |  |  |  |  |
| 0 | Vitvi11g00935\_t001 |  |  |  |  |  |  |  |  |  |  |  |  |
| 0 | Vitvi11g00936\_t001 |  |  |  |  |  |  |  |  |  |  |  |  |
| 0 | Vitvi11g00937\_t001 |  |  |  |  |  |  |  |  |  |  |  |  |
| 0 | Vitvi11g00939\_t001 |  |  |  |  |  |  |  |  |  |  |  |  |
| 0 | Vitvi11g00940\_t001 |  |  |  |  |  |  |  |  |  |  |  |  |
| 0 | Vitvi11g00942\_t001 |  |  |  |  |  |  |  |  |  |  |  |  |
| 0 | Vitvi11g01556\_t001 |  |  |  |  |  |  |  |  |  |  |  |  |
| 0 | Vitvi11g04248\_t001 |  |  |  |  |  |  |  |  |  |  |  |  |
| 0 | Vitvi11g01558\_t001 |  |  |  |  |  |  |  |  |  |  |  |  |
| 0 | Vitvi11g01562\_t001 |  |  |  |  |  |  |  |  |  |  |  |  |
| 0 | Vitvi11g04249\_t001 |  |  |  |  |  |  |  |  |  |  |  |  |
| 0 | Vitvi11g04250\_t001 |  |  |  |  |  |  |  |  |  |  |  |  |
| 0 | Vitvi11g00948\_t001 |  |  |  |  |  |  |  |  |  |  |  |  |
| 0 | Vitvi11g00949\_t002 |  |  |  |  |  |  |  |  |  |  |  |  |
| 0 | Vitvi11g01564\_t001 |  |  |  |  |  |  |  |  |  |  |  |  |
| 0 | Vitvi11g04251\_t001 |  |  |  |  |  |  |  |  |  |  |  |  |
| 0 | Vitvi11g00951\_t001 |  |  |  |  |  |  |  |  |  |  |  |  |
| 0 | Vitvi11g04252\_t001 |  |  |  |  |  |  |  |  |  |  |  |  |
| 0 | Vitvi11g04253\_t001 |  |  |  |  |  |  |  |  |  |  |  |  |
| 0 | Vitvi11g00953\_t001 |  |  |  |  |  |  |  |  |  |  |  |  |
| 0 | Vitvi11g00954\_t002 |  |  |  |  |  |  |  |  |  |  |  |  |
| 0 | Vitvi11g00955\_t001 |  |  |  |  |  |  |  |  |  |  |  |  |
| 0 | Vitvi11g00957\_t001 |  |  |  |  |  |  |  |  |  |  |  |  |
| 0 | Vitvi11g01567\_t001 |  |  |  |  |  |  |  |  |  |  |  |  |
| 0 | Vitvi11g04254\_t001 |  |  |  |  |  |  |  |  |  |  |  |  |
| 0 | Vitvi11g04255\_t001 |  |  |  |  |  |  |  |  |  |  |  |  |
| 0 | Vitvi11g04256\_t001 |  |  |  |  |  |  |  |  |  |  |  |  |
| 0 | Vitvi11g00959\_t001 |  |  |  |  |  |  |  |  |  |  |  |  |
| 0 | Vitvi11g00960\_t001 |  |  |  |  |  |  |  |  |  |  |  |  |
| 0 | Vitvi11g00961\_t001 |  |  |  |  |  |  |  |  |  |  |  |  |
| 0 | Vitvi11g04257\_t001 |  |  |  |  |  |  |  |  |  |  |  |  |
| 0 | Vitvi11g00962\_t001 |  |  |  |  |  |  |  |  |  |  |  |  |
| 0 | Vitvi11g04258\_t001 |  |  |  |  |  |  |  |  |  |  |  |  |
| 0 | Vitvi11g04259\_t001 |  |  |  |  |  |  |  |  |  |  |  |  |
| 0 | Vitvi11g04260\_t001 |  |  |  |  |  |  |  |  |  |  |  |  |
| 0 | Vitvi11g04261\_t001 |  |  |  |  |  |  |  |  |  |  |  |  |
| 0 | Vitvi11g04262\_t001 |  |  |  |  |  |  |  |  |  |  |  |  |
| 0 | Vitvi11g00965\_t001 |  |  |  |  |  |  |  |  |  |  |  |  |
| 0 | Vitvi11g00967\_t001 |  |  |  |  |  |  |  |  |  |  |  |  |
| 0 | Vitvi11g04263\_t001 |  |  |  |  |  |  |  |  |  |  |  |  |
| 0 | Vitvi11g04264\_t001 |  |  |  |  |  |  |  |  |  |  |  |  |
| 0 | Vitvi11g04265\_t001 |  |  |  |  |  |  |  |  |  |  |  |  |
| 0 | Vitvi11g04266\_t001 |  |  |  |  |  |  |  |  |  |  |  |  |
| 0 | Vitvi11g04267\_t001 |  |  |  |  |  |  |  |  |  |  |  |  |
| 0 | Vitvi11g01578\_t001 |  |  |  |  |  |  |  |  |  |  |  |  |
| 0 | Vitvi11g04268\_t001 |  |  |  |  |  |  |  |  |  |  |  |  |
| 0 | Vitvi11g00969\_t003 |  |  |  |  |  |  |  |  |  |  |  |  |
| 0 | Vitvi11g04269\_t001 |  |  |  |  |  |  |  |  |  |  |  |  |
| 0 | Vitvi11g04270\_t001 |  |  |  |  |  |  |  |  |  |  |  |  |
| 0 | Vitvi11g04271\_t001 |  |  |  |  |  |  |  |  |  |  |  |  |
| 0 | Vitvi11g04272\_t001 |  |  |  |  |  |  |  |  |  |  |  |  |
| 0 | Vitvi11g00977\_t001 |  |  |  |  |  |  |  |  |  |  |  |  |
| 0 | Vitvi11g04273\_t001 |  |  |  |  |  |  |  |  |  |  |  |  |
| 0 | Vitvi11g00979\_t001 |  |  |  |  |  |  |  |  |  |  |  |  |
| 0 | Vitvi11g00980\_t001 |  |  |  |  |  |  |  |  |  |  |  |  |
| 0 | Vitvi11g04274\_t001 |  |  |  |  |  |  |  |  |  |  |  |  |
| 0 | Vitvi11g01582\_t001 |  |  |  |  |  |  |  |  |  |  |  |  |
| 0 | Vitvi11g04275\_t001 |  |  |  |  |  |  |  |  |  |  |  |  |
| 0 | Vitvi11g04276\_t001 |  |  |  |  |  |  |  |  |  |  |  |  |
| 0 | Vitvi11g04277\_t001 |  |  |  |  |  |  |  |  |  |  |  |  |
| 0 | Vitvi11g00984\_t001 |  |  |  |  |  |  |  |  |  |  |  |  |
| 0 | Vitvi11g04278\_t001 |  |  |  |  |  |  |  |  |  |  |  |  |
| 0 | Vitvi11g04279\_t001 |  |  |  |  |  |  |  |  |  |  |  |  |
| 0 | Vitvi11g04280\_t001 |  |  |  |  |  |  |  |  |  |  |  |  |
| 0 | Vitvi11g04281\_t001 |  |  |  |  |  |  |  |  |  |  |  |  |
| 0 | Vitvi11g04282\_t001 |  |  |  |  |  |  |  |  |  |  |  |  |
| 0 | Vitvi11g00988\_t001 |  |  |  |  |  |  |  |  |  |  |  |  |
| 0 | Vitvi11g04283\_t001 |  |  |  |  |  |  |  |  |  |  |  |  |
| 0 | Vitvi11g00990\_t001 |  |  |  |  |  |  |  |  |  |  |  |  |
| 0 | Vitvi11g01586\_t001 |  |  |  |  |  |  |  |  |  |  |  |  |
| 0 | Vitvi11g04284\_t001 |  |  |  |  |  |  |  |  |  |  |  |  |
| 0 | Vitvi11g01005\_t001 |  |  |  |  |  |  |  |  |  |  |  |  |
| 0 | Vitvi11g04285\_t001 |  |  |  |  |  |  |  |  |  |  |  |  |
| 0 | Vitvi11g01033\_t001 |  |  |  |  |  |  |  |  |  |  |  |  |
| 0 | Vitvi11g04286\_t001 |  |  |  |  |  |  |  |  |  |  |  |  |
| 0 | Vitvi11g01035\_t001 |  |  |  |  |  |  |  |  |  |  |  |  |
| 0 | Vitvi11g01597\_t001 |  |  |  |  |  |  |  |  |  |  |  |  |
| 0 | Vitvi11g01039\_t001 |  |  |  |  |  |  |  |  |  |  |  |  |
| 0 | Vitvi11g01041\_t001 |  |  |  |  |  |  |  |  |  |  |  |  |
| 0 | Vitvi11g01045\_t001 |  |  |  |  |  |  |  |  |  |  |  |  |
| 0 | Vitvi11g01046\_t001 |  |  |  |  |  |  |  |  |  |  |  |  |
| 0 | Vitvi11g01048\_t001 |  |  |  |  |  |  |  |  |  |  |  |  |
| 0 | Vitvi11g01053\_t001 |  |  |  |  |  |  |  |  |  |  |  |  |
| 0 | Vitvi11g04287\_t001 |  |  |  |  |  |  |  |  |  |  |  |  |
| 0 | Vitvi11g04288\_t001 |  |  |  |  |  |  |  |  |  |  |  |  |
| 0 | Vitvi11g01055\_t001 |  |  |  |  |  |  |  |  |  |  |  |  |
| 0 | Vitvi11g01057\_t001 |  |  |  |  |  |  |  |  |  |  |  |  |
| 0 | Vitvi11g01060\_t001 |  |  |  |  |  |  |  |  |  |  |  |  |
| 0 | Vitvi11g04289\_t001 |  |  |  |  |  |  |  |  |  |  |  |  |
| 0 | Vitvi11g04290\_t001 |  |  |  |  |  |  |  |  |  |  |  |  |
| 0 | Vitvi11g04291\_t001 |  |  |  |  |  |  |  |  |  |  |  |  |
| 0 | Vitvi11g04292\_t001 |  |  |  |  |  |  |  |  |  |  |  |  |
| 0 | Vitvi11g01323\_t001 |  |  |  |  |  |  |  |  |  |  |  |  |
| 0 | Vitvi11g04293\_t001 |  |  |  |  |  |  |  |  |  |  |  |  |
| 0 | Vitvi11g04294\_t001 |  |  |  |  |  |  |  |  |  |  |  |  |
| 0 | Vitvi11g04295\_t001 |  |  |  |  |  |  |  |  |  |  |  |  |
| 0 | Vitvi11g04296\_t001 |  |  |  |  |  |  |  |  |  |  |  |  |
| 0 | Vitvi11g04297\_t001 |  |  |  |  |  |  |  |  |  |  |  |  |
| 0 | Vitvi11g04298\_t001 |  |  |  |  |  |  |  |  |  |  |  |  |
| 0 | Vitvi11g04299\_t001 |  |  |  |  |  |  |  |  |  |  |  |  |
| 0 | Vitvi11g04300\_t001 |  |  |  |  |  |  |  |  |  |  |  |  |
| 0 | Vitvi11g01072\_t001 |  |  |  |  |  |  |  |  |  |  |  |  |
| 0 | Vitvi11g01073\_t001 |  |  |  |  |  |  |  |  |  |  |  |  |
| 0 | Vitvi11g01075\_t001 |  |  |  |  |  |  |  |  |  |  |  |  |
| 0 | Vitvi11g04301\_t001 |  |  |  |  |  |  |  |  |  |  |  |  |
| 0 | Vitvi11g01083\_t001 |  |  |  |  |  |  |  |  |  |  |  |  |
| 0 | Vitvi11g01084\_t001 |  |  |  |  |  |  |  |  |  |  |  |  |
| 0 | Vitvi11g04302\_t001 |  |  |  |  |  |  |  |  |  |  |  |  |
| 0 | Vitvi11g04303\_t001 |  |  |  |  |  |  |  |  |  |  |  |  |
| 0 | Vitvi11g01604\_t001 |  |  |  |  |  |  |  |  |  |  |  |  |
| 0 | Vitvi11g01605\_t001 |  |  |  |  |  |  |  |  |  |  |  |  |
| 0 | Vitvi11g01085\_t001 |  |  |  |  |  |  |  |  |  |  |  |  |
| 0 | Vitvi11g01086\_t001 |  |  |  |  |  |  |  |  |  |  |  |  |
| 0 | Vitvi11g04304\_t001 |  |  |  |  |  |  |  |  |  |  |  |  |
| 0 | Vitvi11g01606\_t001 |  |  |  |  |  |  |  |  |  |  |  |  |
| 0 | Vitvi11g01093\_t001 |  |  |  |  |  |  |  |  |  |  |  |  |
| 0 | Vitvi11g01094\_t001 |  |  |  |  |  |  |  |  |  |  |  |  |
| 0 | Vitvi11g04305\_t001 |  |  |  |  |  |  |  |  |  |  |  |  |
| 0 | Vitvi11g01098\_t001 |  |  |  |  |  |  |  |  |  |  |  |  |
| 0 | Vitvi11g01099\_t001 |  |  |  |  |  |  |  |  |  |  |  |  |
| 0 | Vitvi11g01100\_t001 |  |  |  |  |  |  |  |  |  |  |  |  |
| 0 | Vitvi11g01101\_t001 |  |  |  |  |  |  |  |  |  |  |  |  |
| 0 | Vitvi11g01102\_t001 |  |  |  |  |  |  |  |  |  |  |  |  |
| 0 | Vitvi11g01104\_t001 |  |  |  |  |  |  |  |  |  |  |  |  |
| 0 | Vitvi11g01608\_t001 |  |  |  |  |  |  |  |  |  |  |  |  |
| 0 | Vitvi11g01105\_t001 |  |  |  |  |  |  |  |  |  |  |  |  |
| 0 | Vitvi11g01106\_t001 |  |  |  |  |  |  |  |  |  |  |  |  |
| 0 | Vitvi11g01107\_t002 |  |  |  |  |  |  |  |  |  |  |  |  |
| 0 | Vitvi11g01110\_t001 |  |  |  |  |  |  |  |  |  |  |  |  |
| 0 | Vitvi11g04306\_t001 |  |  |  |  |  |  |  |  |  |  |  |  |
| 0 | Vitvi11g04307\_t002 |  |  |  |  |  |  |  |  |  |  |  |  |
| 0 | Vitvi11g04308\_t001 |  |  |  |  |  |  |  |  |  |  |  |  |
| 0 | Vitvi11g04309\_t001 |  |  |  |  |  |  |  |  |  |  |  |  |
| 0 | Vitvi11g01117\_t001 |  |  |  |  |  |  |  |  |  |  |  |  |
| 0 | Vitvi11g01118\_t001 |  |  |  |  |  |  |  |  |  |  |  |  |
| 0 | Vitvi11g01119\_t001 |  |  |  |  |  |  |  |  |  |  |  |  |
| 0 | Vitvi11g01620\_t001 |  |  |  |  |  |  |  |  |  |  |  |  |
| 0 | Vitvi11g04310\_t001 |  |  |  |  |  |  |  |  |  |  |  |  |
| 0 | Vitvi11g01621\_t001 |  |  |  |  |  |  |  |  |  |  |  |  |
| 0 | Vitvi11g04311\_t001 |  |  |  |  |  |  |  |  |  |  |  |  |
| 0 | Vitvi11g01128\_t001 |  |  |  |  |  |  |  |  |  |  |  |  |
| 0 | Vitvi11g04312\_t001 |  |  |  |  |  |  |  |  |  |  |  |  |
| 0 | Vitvi11g04313\_t001 |  |  |  |  |  |  |  |  |  |  |  |  |
| 0 | Vitvi11g01130\_t001 |  |  |  |  |  |  |  |  |  |  |  |  |
| 0 | Vitvi11g01623\_t001 |  |  |  |  |  |  |  |  |  |  |  |  |
| 0 | Vitvi11g01132\_t001 |  |  |  |  |  |  |  |  |  |  |  |  |
| 0 | Vitvi11g01133\_t002 |  |  |  |  |  |  |  |  |  |  |  |  |
| 0 | Vitvi11g01134\_t006 |  |  |  |  |  |  |  |  |  |  |  |  |
| 0 | Vitvi11g04314\_t001 |  |  |  |  |  |  |  |  |  |  |  |  |
| 0 | Vitvi11g01624\_t001 |  |  |  |  |  |  |  |  |  |  |  |  |
| 0 | Vitvi11g04315\_t001 |  |  |  |  |  |  |  |  |  |  |  |  |
| 0 | Vitvi11g01136\_t001 |  |  |  |  |  |  |  |  |  |  |  |  |
| 0 | Vitvi11g01137\_t001 |  |  |  |  |  |  |  |  |  |  |  |  |
| 0 | Vitvi11g01138\_t002 |  |  |  |  |  |  |  |  |  |  |  |  |
| 0 | Vitvi11g01139\_t001 |  |  |  |  |  |  |  |  |  |  |  |  |
| 0 | Vitvi11g04316\_t001 |  |  |  |  |  |  |  |  |  |  |  |  |
| 0 | Vitvi11g01140\_t001 |  |  |  |  |  |  |  |  |  |  |  |  |
| 0 | Vitvi11g04317\_t001 |  |  |  |  |  |  |  |  |  |  |  |  |
| 0 | Vitvi11g01141\_t001 |  |  |  |  |  |  |  |  |  |  |  |  |
| 0 | Vitvi11g04318\_t001 |  |  |  |  |  |  |  |  |  |  |  |  |
| 0 | Vitvi11g01627\_t001 |  |  |  |  |  |  |  |  |  |  |  |  |
| 0 | Vitvi11g01142\_t001 |  |  |  |  |  |  |  |  |  |  |  |  |
| 0 | Vitvi11g01143\_t001 |  |  |  |  |  |  |  |  |  |  |  |  |
| 0 | Vitvi11g04319\_t001 |  |  |  |  |  |  |  |  |  |  |  |  |
| 0 | Vitvi11g01144\_t001 |  |  |  |  |  |  |  |  |  |  |  |  |
| 0 | Vitvi11g01145\_t001 |  |  |  |  |  |  |  |  |  |  |  |  |
| 0 | Vitvi11g01146\_t001 |  |  |  |  |  |  |  |  |  |  |  |  |
| 0 | Vitvi11g04320\_t001 |  |  |  |  |  |  |  |  |  |  |  |  |
| 0 | Vitvi11g01147\_t001 |  |  |  |  |  |  |  |  |  |  |  |  |
| 0 | Vitvi11g01148\_t001 |  |  |  |  |  |  |  |  |  |  |  |  |
| 0 | Vitvi11g01150\_t001 |  |  |  |  |  |  |  |  |  |  |  |  |
| 0 | Vitvi11g01151\_t001 |  |  |  |  |  |  |  |  |  |  |  |  |
| 0 | Vitvi11g01153\_t001 |  |  |  |  |  |  |  |  |  |  |  |  |
| 0 | Vitvi11g04321\_t001 |  |  |  |  |  |  |  |  |  |  |  |  |
| 0 | Vitvi11g01155\_t001 |  |  |  |  |  |  |  |  |  |  |  |  |
| 0 | Vitvi11g01156\_t001 |  |  |  |  |  |  |  |  |  |  |  |  |
| 0 | Vitvi11g01158\_t001 |  |  |  |  |  |  |  |  |  |  |  |  |
| 0 | Vitvi11g01159\_t001 |  |  |  |  |  |  |  |  |  |  |  |  |
| 0 | Vitvi11g01160\_t001 |  |  |  |  |  |  |  |  |  |  |  |  |
| 0 | Vitvi11g01162\_t001 |  |  |  |  |  |  |  |  |  |  |  |  |
| 0 | Vitvi11g01163\_t002 |  |  |  |  |  |  |  |  |  |  |  |  |
| 0 | Vitvi11g01629\_t001 |  |  |  |  |  |  |  |  |  |  |  |  |
| 0 | Vitvi11g01165\_t001 |  |  |  |  |  |  |  |  |  |  |  |  |
| 0 | Vitvi11g01630\_t003 |  |  |  |  |  |  |  |  |  |  |  |  |
| 0 | Vitvi11g01632\_t001 |  |  |  |  |  |  |  |  |  |  |  |  |
| 0 | Vitvi11g01633\_t001 |  |  |  |  |  |  |  |  |  |  |  |  |
| 0 | Vitvi11g04322\_t001 |  |  |  |  |  |  |  |  |  |  |  |  |
| 0 | Vitvi11g01634\_t001 |  |  |  |  |  |  |  |  |  |  |  |  |
| 0 | Vitvi11g04323\_t001 |  |  |  |  |  |  |  |  |  |  |  |  |
| 0 | Vitvi11g01635\_t001 |  |  |  |  |  |  |  |  |  |  |  |  |
| 0 | Vitvi11g04324\_t001 |  |  |  |  |  |  |  |  |  |  |  |  |
| 0 | Vitvi11g01636\_t001 |  |  |  |  |  |  |  |  |  |  |  |  |
| 0 | Vitvi11g01637\_t001 |  |  |  |  |  |  |  |  |  |  |  |  |
| 0 | Vitvi11g01638\_t001 |  |  |  |  |  |  |  |  |  |  |  |  |
| 0 | Vitvi11g01168\_t001 |  |  |  |  |  |  |  |  |  |  |  |  |
| 0 | Vitvi11g04325\_t001 |  |  |  |  |  |  |  |  |  |  |  |  |
| 0 | Vitvi11g01169\_t001 |  |  |  |  |  |  |  |  |  |  |  |  |
| 0 | Vitvi11g04326\_t001 |  |  |  |  |  |  |  |  |  |  |  |  |
| 0 | Vitvi11g01173\_t001 |  |  |  |  |  |  |  |  |  |  |  |  |
| 0 | Vitvi11g01176\_t001 |  |  |  |  |  |  |  |  |  |  |  |  |
| 0 | Vitvi11g01177\_t001 |  |  |  |  |  |  |  |  |  |  |  |  |
| 0 | Vitvi11g04327\_t001 |  |  |  |  |  |  |  |  |  |  |  |  |
| 0 | Vitvi11g04328\_t001 |  |  |  |  |  |  |  |  |  |  |  |  |
| 0 | Vitvi11g01179\_t001 |  |  |  |  |  |  |  |  |  |  |  |  |
| 0 | Vitvi11g01641\_t001 |  |  |  |  |  |  |  |  |  |  |  |  |
| 0 | Vitvi11g01642\_t001 |  |  |  |  |  |  |  |  |  |  |  |  |
| 0 | Vitvi11g01180\_t001 |  |  |  |  |  |  |  |  |  |  |  |  |
| 0 | Vitvi11g01181\_t001 |  |  |  |  |  |  |  |  |  |  |  |  |
| 0 | Vitvi11g01182\_t001 |  |  |  |  |  |  |  |  |  |  |  |  |
| 0 | Vitvi11g01183\_t001 |  |  |  |  |  |  |  |  |  |  |  |  |
| 0 | Vitvi11g01184\_t001 |  |  |  |  |  |  |  |  |  |  |  |  |
| 0 | Vitvi11g01643\_t001 |  |  |  |  |  |  |  |  |  |  |  |  |
| 0 | Vitvi11g01185\_t001 |  |  |  |  |  |  |  |  |  |  |  |  |
| 0 | Vitvi11g01186\_t001 |  |  |  |  |  |  |  |  |  |  |  |  |
| 0 | Vitvi11g01188\_t001 |  |  |  |  |  |  |  |  |  |  |  |  |
| 0 | Vitvi11g04329\_t001 |  |  |  |  |  |  |  |  |  |  |  |  |
| 0 | Vitvi11g01644\_t001 |  |  |  |  |  |  |  |  |  |  |  |  |
| 0 | Vitvi11g01191\_t001 |  |  |  |  |  |  |  |  |  |  |  |  |
| 0 | Vitvi11g01645\_t001 |  |  |  |  |  |  |  |  |  |  |  |  |
| 0 | Vitvi11g04330\_t001 |  |  |  |  |  |  |  |  |  |  |  |  |
| 0 | Vitvi11g01192\_t001 |  |  |  |  |  |  |  |  |  |  |  |  |
| 0 | Vitvi11g01195\_t001 |  |  |  |  |  |  |  |  |  |  |  |  |
| 0 | Vitvi11g01197\_t001 |  |  |  |  |  |  |  |  |  |  |  |  |
| 0 | Vitvi11g04331\_t001 |  |  |  |  |  |  |  |  |  |  |  |  |
| 0 | Vitvi11g01198\_t001 |  |  |  |  |  |  |  |  |  |  |  |  |
| 0 | Vitvi11g01199\_t001 |  |  |  |  |  |  |  |  |  |  |  |  |
| 0 | Vitvi11g01200\_t001 |  |  |  |  |  |  |  |  |  |  |  |  |
| 0 | Vitvi11g01201\_t001 |  |  |  |  |  |  |  |  |  |  |  |  |
| 0 | Vitvi11g01202\_t001 |  |  |  |  |  |  |  |  |  |  |  |  |
| 0 | Vitvi11g01203\_t001 |  |  |  |  |  |  |  |  |  |  |  |  |
| 0 | Vitvi11g01205\_t001 |  |  |  |  |  |  |  |  |  |  |  |  |
| 0 | Vitvi11g04332\_t001 |  |  |  |  |  |  |  |  |  |  |  |  |
| 0 | Vitvi11g04333\_t001 |  |  |  |  |  |  |  |  |  |  |  |  |
| 0 | Vitvi11g04334\_t001 |  |  |  |  |  |  |  |  |  |  |  |  |
| 0 | Vitvi11g01208\_t001 |  |  |  |  |  |  |  |  |  |  |  |  |
| 0 | Vitvi11g01650\_t002 |  |  |  |  |  |  |  |  |  |  |  |  |
| 0 | Vitvi11g01209\_t001 |  |  |  |  |  |  |  |  |  |  |  |  |
| 0 | Vitvi11g04335\_t001 |  |  |  |  |  |  |  |  |  |  |  |  |
| 0 | Vitvi11g01210\_t001 |  |  |  |  |  |  |  |  |  |  |  |  |
| 0 | Vitvi11g01211\_t001 |  |  |  |  |  |  |  |  |  |  |  |  |
| 0 | Vitvi11g01212\_t001 |  |  |  |  |  |  |  |  |  |  |  |  |
| 0 | Vitvi11g01651\_t001 |  |  |  |  |  |  |  |  |  |  |  |  |
| 0 | Vitvi11g01652\_t001 |  |  |  |  |  |  |  |  |  |  |  |  |
| 0 | Vitvi11g01213\_t001 |  |  |  |  |  |  |  |  |  |  |  |  |
| 0 | Vitvi11g04336\_t001 |  |  |  |  |  |  |  |  |  |  |  |  |
| 0 | Vitvi11g01220\_t001 |  |  |  |  |  |  |  |  |  |  |  |  |
| 0 | Vitvi11g04337\_t001 |  |  |  |  |  |  |  |  |  |  |  |  |
| 0 | Vitvi11g01222\_t001 |  |  |  |  |  |  |  |  |  |  |  |  |
| 0 | Vitvi11g04338\_t001 |  |  |  |  |  |  |  |  |  |  |  |  |
| 0 | Vitvi11g01224\_t001 |  |  |  |  |  |  |  |  |  |  |  |  |
| 0 | Vitvi11g01227\_t001 |  |  |  |  |  |  |  |  |  |  |  |  |
| 0 | Vitvi11g01228\_t001 |  |  |  |  |  |  |  |  |  |  |  |  |
| 0 | Vitvi11g01656\_t001 |  |  |  |  |  |  |  |  |  |  |  |  |
| 0 | Vitvi11g01657\_t001 |  |  |  |  |  |  |  |  |  |  |  |  |
| 0 | Vitvi11g01230\_t001 |  |  |  |  |  |  |  |  |  |  |  |  |
| 0 | Vitvi11g01231\_t001 |  |  |  |  |  |  |  |  |  |  |  |  |
| 0 | Vitvi11g01232\_t001 |  |  |  |  |  |  |  |  |  |  |  |  |
| 0 | Vitvi11g04339\_t001 |  |  |  |  |  |  |  |  |  |  |  |  |
| 0 | Vitvi11g01233\_t001 |  |  |  |  |  |  |  |  |  |  |  |  |
| 0 | Vitvi11g01235\_t001 |  |  |  |  |  |  |  |  |  |  |  |  |
| 0 | Vitvi11g01236\_t001 |  |  |  |  |  |  |  |  |  |  |  |  |
| 0 | Vitvi11g04340\_t001 |  |  |  |  |  |  |  |  |  |  |  |  |
| 0 | Vitvi11g01238\_t002 |  |  |  |  |  |  |  |  |  |  |  |  |
| 0 | Vitvi11g04341\_t001 |  |  |  |  |  |  |  |  |  |  |  |  |
| 0 | Vitvi11g04342\_t001 |  |  |  |  |  |  |  |  |  |  |  |  |
| 0 | Vitvi11g01658\_t001 |  |  |  |  |  |  |  |  |  |  |  |  |
| 0 | Vitvi11g01239\_t001 |  |  |  |  |  |  |  |  |  |  |  |  |
| 0 | Vitvi11g01240\_t001 |  |  |  |  |  |  |  |  |  |  |  |  |
| 0 | Vitvi11g01241\_t001 |  |  |  |  |  |  |  |  |  |  |  |  |
| 0 | Vitvi11g04343\_t001 |  |  |  |  |  |  |  |  |  |  |  |  |
| 0 | Vitvi11g04344\_t001 |  |  |  |  |  |  |  |  |  |  |  |  |
| 0 | Vitvi11g01243\_t001 |  |  |  |  |  |  |  |  |  |  |  |  |
| 0 | Vitvi11g01244\_t001 |  |  |  |  |  |  |  |  |  |  |  |  |
| 0 | Vitvi11g04345\_t001 |  |  |  |  |  |  |  |  |  |  |  |  |
| 0 | Vitvi11g04346\_t001 |  |  |  |  |  |  |  |  |  |  |  |  |
| 0 | Vitvi11g04347\_t001 |  |  |  |  |  |  |  |  |  |  |  |  |
| 0 | Vitvi11g01250\_t001 |  |  |  |  |  |  |  |  |  |  |  |  |
| 0 | Vitvi11g04348\_t001 |  |  |  |  |  |  |  |  |  |  |  |  |
| 0 | Vitvi11g01251\_t001 |  |  |  |  |  |  |  |  |  |  |  |  |
| 0 | Vitvi11g01662\_t001 |  |  |  |  |  |  |  |  |  |  |  |  |
| 0 | Vitvi11g01663\_t001 |  |  |  |  |  |  |  |  |  |  |  |  |
| 0 | Vitvi11g04349\_t001 |  |  |  |  |  |  |  |  |  |  |  |  |
| 0 | Vitvi11g01253\_t001 |  |  |  |  |  |  |  |  |  |  |  |  |
| 0 | Vitvi11g01664\_t001 |  |  |  |  |  |  |  |  |  |  |  |  |
| 0 | Vitvi11g01665\_t001 |  |  |  |  |  |  |  |  |  |  |  |  |
| 0 | Vitvi11g01255\_t001 |  |  |  |  |  |  |  |  |  |  |  |  |
| 0 | Vitvi11g01667\_t001 |  |  |  |  |  |  |  |  |  |  |  |  |
| 0 | Vitvi11g04350\_t001 |  |  |  |  |  |  |  |  |  |  |  |  |
| 0 | Vitvi11g01668\_t001 |  |  |  |  |  |  |  |  |  |  |  |  |
| 0 | Vitvi11g01257\_t001 |  |  |  |  |  |  |  |  |  |  |  |  |
| 0 | Vitvi11g04351\_t001 |  |  |  |  |  |  |  |  |  |  |  |  |
| 0 | Vitvi11g04352\_t001 |  |  |  |  |  |  |  |  |  |  |  |  |
| 0 | Vitvi11g01258\_t001 |  |  |  |  |  |  |  |  |  |  |  |  |
| 0 | Vitvi11g01259\_t001 |  |  |  |  |  |  |  |  |  |  |  |  |
| 0 | Vitvi11g01260\_t003 |  |  |  |  |  |  |  |  |  |  |  |  |
| 0 | Vitvi11g01671\_t001 |  |  |  |  |  |  |  |  |  |  |  |  |
| 0 | Vitvi11g04353\_t001 |  |  |  |  |  |  |  |  |  |  |  |  |
| 0 | Vitvi11g01261\_t001 |  |  |  |  |  |  |  |  |  |  |  |  |
| 0 | Vitvi11g04354\_t001 |  |  |  |  |  |  |  |  |  |  |  |  |
| 0 | Vitvi11g04355\_t001 |  |  |  |  |  |  |  |  |  |  |  |  |
| 0 | Vitvi11g01673\_t001 |  |  |  |  |  |  |  |  |  |  |  |  |
| 0 | Vitvi11g01674\_t001 |  |  |  |  |  |  |  |  |  |  |  |  |
| 0 | Vitvi11g01263\_t001 |  |  |  |  |  |  |  |  |  |  |  |  |
| 0 | Vitvi11g01675\_t001 |  |  |  |  |  |  |  |  |  |  |  |  |
| 0 | Vitvi11g04356\_t001 |  |  |  |  |  |  |  |  |  |  |  |  |
| 0 | Vitvi11g01265\_t001 |  |  |  |  |  |  |  |  |  |  |  |  |
| 0 | Vitvi11g01266\_t001 |  |  |  |  |  |  |  |  |  |  |  |  |
| 0 | Vitvi11g01267\_t001 |  |  |  |  |  |  |  |  |  |  |  |  |
| 0 | Vitvi11g01268\_t001 |  |  |  |  |  |  |  |  |  |  |  |  |
| 0 | Vitvi11g01676\_t001 |  |  |  |  |  |  |  |  |  |  |  |  |
| 0 | Vitvi11g01677\_t001 |  |  |  |  |  |  |  |  |  |  |  |  |
| 0 | Vitvi11g04357\_t001 |  |  |  |  |  |  |  |  |  |  |  |  |
| 0 | Vitvi11g01681\_t001 |  |  |  |  |  |  |  |  |  |  |  |  |
| 0 | Vitvi11g01682\_t001 |  |  |  |  |  |  |  |  |  |  |  |  |
| 0 | Vitvi11g04358\_t001 |  |  |  |  |  |  |  |  |  |  |  |  |
| 0 | Vitvi11g01684\_t001 |  |  |  |  |  |  |  |  |  |  |  |  |
| 0 | Vitvi11g01269\_t001 |  |  |  |  |  |  |  |  |  |  |  |  |
| 0 | Vitvi11g04359\_t001 |  |  |  |  |  |  |  |  |  |  |  |  |
| 0 | Vitvi11g01271\_t001 |  |  |  |  |  |  |  |  |  |  |  |  |
| 0 | Vitvi11g01272\_t001 |  |  |  |  |  |  |  |  |  |  |  |  |
| 0 | Vitvi11g01273\_t001 |  |  |  |  |  |  |  |  |  |  |  |  |
| 0 | Vitvi11g01277\_t001 |  |  |  |  |  |  |  |  |  |  |  |  |
| 0 | Vitvi11g01685\_t001 |  |  |  |  |  |  |  |  |  |  |  |  |
| 0 | Vitvi11g01686\_t001 |  |  |  |  |  |  |  |  |  |  |  |  |
| 0 | Vitvi11g01687\_t001 |  |  |  |  |  |  |  |  |  |  |  |  |
| 0 | Vitvi11g04360\_t001 |  |  |  |  |  |  |  |  |  |  |  |  |
| 0 | Vitvi11g01279\_t001 |  |  |  |  |  |  |  |  |  |  |  |  |
| 0 | Vitvi11g01281\_t001 |  |  |  |  |  |  |  |  |  |  |  |  |
| 0 | Vitvi11g01688\_t001 |  |  |  |  |  |  |  |  |  |  |  |  |
| 0 | Vitvi11g01282\_t001 |  |  |  |  |  |  |  |  |  |  |  |  |
| 0 | Vitvi11g04361\_t001 |  |  |  |  |  |  |  |  |  |  |  |  |
| 0 | Vitvi11g04362\_t001 |  |  |  |  |  |  |  |  |  |  |  |  |
| 0 | Vitvi11g01283\_t001 |  |  |  |  |  |  |  |  |  |  |  |  |
| 0 | Vitvi11g01284\_t001 |  |  |  |  |  |  |  |  |  |  |  |  |
| 0 | Vitvi11g01285\_t001 |  |  |  |  |  |  |  |  |  |  |  |  |
| 0 | Vitvi11g01286\_t001 |  |  |  |  |  |  |  |  |  |  |  |  |
| 0 | Vitvi11g01691\_t001 |  |  |  |  |  |  |  |  |  |  |  |  |
| 0 | Vitvi11g01692\_t001 |  |  |  |  |  |  |  |  |  |  |  |  |
| 0 | Vitvi11g01693\_t001 |  |  |  |  |  |  |  |  |  |  |  |  |
| 0 | Vitvi11g01287\_t001 |  |  |  |  |  |  |  |  |  |  |  |  |
| 0 | Vitvi11g04363\_t001 |  |  |  |  |  |  |  |  |  |  |  |  |
| 0 | Vitvi11g04364\_t001 |  |  |  |  |  |  |  |  |  |  |  |  |
| 0 | Vitvi11g01290\_t001 |  |  |  |  |  |  |  |  |  |  |  |  |
| 0 | Vitvi11g01291\_t001 |  |  |  |  |  |  |  |  |  |  |  |  |
| 0 | Vitvi11g01293\_t001 |  |  |  |  |  |  |  |  |  |  |  |  |
| 0 | Vitvi11g01294\_t001 |  |  |  |  |  |  |  |  |  |  |  |  |
| 0 | Vitvi11g04365\_t001 |  |  |  |  |  |  |  |  |  |  |  |  |
| 0 | Vitvi11g01295\_t001 |  |  |  |  |  |  |  |  |  |  |  |  |
| 0 | Vitvi11g01296\_t001 |  |  |  |  |  |  |  |  |  |  |  |  |
| 0 | Vitvi11g01297\_t001.1.6037826a |  |  |  |  |  |  |  |  |  |  |  |  |
| 0 | Vitvi11g01298\_t002 |  |  |  |  |  |  |  |  |  |  |  |  |
| 0 | Vitvi11g01300\_t002 |  |  |  |  |  |  |  |  |  |  |  |  |
| 0 | Vitvi11g04366\_t001 |  |  |  |  |  |  |  |  |  |  |  |  |
| 0 | Vitvi11g01301\_t001 |  |  |  |  |  |  |  |  |  |  |  |  |
| 0 | Vitvi11g01303\_t001 |  |  |  |  |  |  |  |  |  |  |  |  |
| 0 | Vitvi11g04367\_t001 |  |  |  |  |  |  |  |  |  |  |  |  |
| 0 | Vitvi11g04368\_t001 |  |  |  |  |  |  |  |  |  |  |  |  |
| 0 | Vitvi11g01307\_t001 |  |  |  |  |  |  |  |  |  |  |  |  |
| 0 | Vitvi11g01308\_t001 |  |  |  |  |  |  |  |  |  |  |  |  |
| 0 | Vitvi11g01309\_t001 |  |  |  |  |  |  |  |  |  |  |  |  |
| 0 | Vitvi11g01310\_t001 |  |  |  |  |  |  |  |  |  |  |  |  |
| 0 | Vitvi11g01311\_t001 |  |  |  |  |  |  |  |  |  |  |  |  |
| 0 | Vitvi11g01702\_t001 |  |  |  |  |  |  |  |  |  |  |  |  |
| 0 | Vitvi11g01316\_t001 |  |  |  |  |  |  |  |  |  |  |  |  |
| 0 | Vitvi11g04369\_t001 |  |  |  |  |  |  |  |  |  |  |  |  |
| 0 | Vitvi11g01318\_t001 |  |  |  |  |  |  |  |  |  |  |  |  |
| 0 | Vitvi11g01319\_t002 |  |  |  |  |  |  |  |  |  |  |  |  |
